# Assessment of Tissue Specific Distribution and Seasonal Variation of Alkaloids in *Alstonia scholaris*

**DOI:** 10.3390/metabo12070607

**Published:** 2022-06-30

**Authors:** Rohit Mahar, Nagarajan Manivel, Sanjeev Kanojiya, Dipak K. Mishra, Sanjeev K. Shukla

**Affiliations:** Sophisticated Analytical Instrument Facility and Research, CSIR-Central Drug Research Institute, Lucknow 226031, India; rohitmahar4u@gmail.com (R.M.); nagambt@gmail.com (N.M.); sanjeev_kanojiya@cdri.res.in (S.K.); dk_mishra@cdri.res.in (D.K.M.)

**Keywords:** metabolic profiling, multivariate analysis, clustering analysis, quantitative analysis, chemical shift

## Abstract

*Alstonia scholaris* is a well-known source of alkaloids and widely recognized for therapeutic purposes to treat the ailments in human and livestock. However, the composition and production of alkaloids vary due to tissue specific metabolism and seasonal variation. This study investigated alkaloids in leaves, stems, trunk barks, fruits, and flowers of *A. scholaris*. The impact of seasonal changes on the production of alkaloids in the leaves of *A. scholaris* was also investigated. One and two-dimensional Nuclear Magnetic Resonance (NMR) experiments were utilized for the characterization of alkaloids and total eight alkaloids (picrinine, picralinal, akuammidine, 19 *S* scholaricine, 19,20 *E* vallesamine, Nb-demethylalstogustine N-Oxide, Nb-demethylalstogustine, and echitamine) were characterized and quantified. Quantitative and multivariate analysis suggested that the alkaloids content is tissue specific, illustrating the effect of plant tissue organization on alkaloidal production in *A. scholaris*. The results suggest that the best part to obtain alkaloids is trunk barks, since it contains 7 alkaloids. However, the best part for isolating picrinine, picralinal, akuammidine, 19 *S* scholaricine, and 19,20 *E* vallesamine is fruit, since it shows highest amount of these alkaloids. Undoubtedly, NMR and statistical methods are very helpful to differentiate the profile of alkaloids in *A. scholaris*.

## 1. Introduction

*Alstonia scholaris* belongs to the family Apocynaceae and is well known as Devil’s tree or Saptparni. It is widely distributed in Asia especially in India, China and Malaysia [1]. It has been used as a traditional medicine for the treatment of various human and livestock diseases and several biologically active compounds have been isolated from this plant [2,3]. *Alstonia scholaris* (AS) is a very rich source of alkaloids, terpenoids and flavonoids etc. There is therefore great interest among scientific groups to explore this medicinal plant for therapeutic applications. Plants have been a great source of secondary metabolites. The presence and quantity of secondary metabolites depends upon the specific part or tissues of the plant, and seasonal variation of the particular region where the plant is grown. Different chemical composition and variable production of secondary metabolites among different geographical regions and seasons have been well documented for terrestrial plants [4]. Herbal medicine is an integral part of drug discovery and development, but due to the complexity of phytoconstituents, the methodology of choice for the identification of phytoconstituents in herbal medicine is mainly to get a characteristic fingerprint of a plant part, that represents the inherent chemical information of the plant species. It has been recognized that a slight variation in the metabolome of plants could be explained by the perturbations imposed on plants. These perturbations might include environmental, physical, abiotic, nutritional stresses and several prolonged mutations. The term “metabolome” refers to the observable chemical profile of the metabolites in a sample [5]. Metabolic profiling would be more useful alongside a wide-spectrum chemical analysis, which is rapid, non-invasive, highly reproducible, and stable with time , while requiring minimal sample preparation. Among many analytical techniques for metabolic profiling, proton (^1^H) NMR spectroscopy is an excellent technique which meets such demands [6]. In plant systems, metabolome-related changes can be observed at metabolite levels which are closely associated with climatic condition, yield, disease, stress resistance, nutritional traits, and genetic functionality. Metabolic profiling has been extensively utilized on a wide range of plants including tobacco [7], tea [8], grapes [9], tomatoes [10], and wheat [11] using a variety of analytical techniques. The NMR and multivariate statistical method have been applied for the metabolic profiling of various kinds of wine [12], coffees [13], juices [14], and beer [15], suggesting a wide range of applications of NMR and chemometric analysis.

Alkaloids stand as a major class of compounds, which is very important in the development of new biologically active principles because of the great diversity in chemical structures and pharmacological properties [16,17,18]. Several alkaloids, especially indole alkaloids, have been isolated from the trunk bark, root, leaves and flowers of *Alstonia* species [19,20,21]. Leaves of AS collected from Indian subcontinent showed a diverse alkaloidal pattern [22,23,24] and picrinine [25], picralinal [26], and 19,20 *E* vallesamine have been identified from this part [27]. Investigation of AS has shown that the major alkaloid obtained from the barks was echitamine, which is the most important alkaloids present in this plant [28]. The bark of this plant has also been investigated for alkaloids, including akuammiginone, echitamidine-N-oxide, 19-O-α-D-glucopyranoside, echitaminic acid, echitamidine N-oxide, Nb-demethylalstogustine N-oxide, akuammicine N-Oxide and Nb-demethylalstogustine [29]. Fruits of AS were investigated for alkaloids and found to contain akuammidine and 19 *S* scholaricine [19]. However, isolating every single secondary metabolites from a plant extract is not always possible due to the requirement of highly advanced preparative high performance liquid chromatography [30] and the complex chemistry of the compounds [31]. For this reason, the analysis of bioactive compounds in the mixtures (or extracts) is a high priority as synergism of the compounds can be sustained in the extracts [32]. NMR spectroscopy is a widely utilized tool for the structure characterization of natural products [33] and is unbiased since the results do not depend on the ionization conditions as in Mass Spectrometry [34]. Additionally, NMR can be used to elucidate the complex/unprecedented compounds [35] and metabolites in a complex plant extract [36,37].

The objectives of this study are: (1) Method development of the identification of alkaloids, employing extensive NMR experiments in the extracts of AS. Previous studies isolated and characterized alkaloids [22,26,29], but the method proposed here provides direct identification and quantitation of alkaloids in the extracts of AS. (2) Metabolic profiling of alkaloids in leaves, fruits, flowers, stems, and trunk barks of AS, using the combination of NMR spectroscopy and chemometric multivariate statistical analysis.

## 2. Results

### 2.1. Identification of Alkaloids in AS

The ^1^H NMR spectra of ethanolic extract of the leaves of AS were recorded in various solvents such as Acetone-*d*_6_, CDCl_3_, MeOD-*d*_4_ and DMSO-*d*_6_, but could not detect alkaloid signals (Supplementary Figure S1). The ^1^H NMR spectra were recorded for all of the three fractions of ethanolic extract and spectra were evaluated. Ethyl acetate-II (AS-EtOAc-II) fraction showed the signals for alkaloids (Supplementary Figure S2), so the ethyl acetate-II (AS-EtOAc-II) fraction was further used for the NMR analysis of all of the samples.

**Figure 1:**
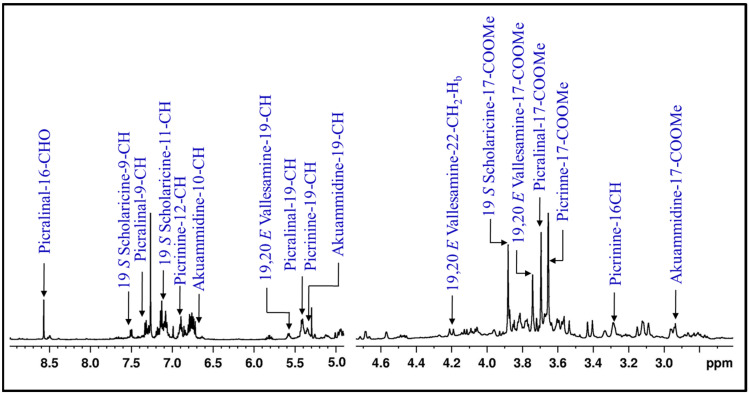
**Profiling of alkaloids in AS:** Representative ^1^H NMR spectrum of AS−FR (AS−fruits) sample demonstrating the characteristics NMR resonances of alkaloids.

The alkaloids were characterized with the help of 1D and 2D NMR experiments in all of the parts of AS. In fruit samples, ^1^H NMR signals for picrinine, 19 *S* scholaricine and 19,20 *E* vallesamine were observed, along with signals at 3.69 ppm (s) for 17-COOMe and 8.56 ppm (s) for 16-CHO, which indicated the presence of picralinal (Figure 1). HSQC NMR spectrum showed a ^1^H-^13^C correlation between 3.69 and 52.2 ppm and also between 8.56 and 197.7 ppm (Supplementary Figure S3). HMBC correlations between 3.69 (17-COOMe) and 168.06 ppm (17-CO), and also the correlation of 16-CHO with 31.4 (C-15) and 65.1 ppm (C-16), confirmed the presence of picralinal. ^1^H NMR signal at 2.94 ppm was also observed, which correlates with a ^13^C signal at 51.3 ppm in HSQC spectrum. HMBC correlations were observed between 2.94 (17-COOMe) and 173.8 ppm and between 3.11 (21-CHa) and 3.80 ppm (21-CHb) to 82.0 ppm (5C), thereby establishing the presence of akuammidine in the fruits of AS (Supplementary Figure S4).

The HSQC NMR spectrum showed a correlation between 3.88 and 52.1 ppm and fixed the carbon position for 17-COOMe at 52.1 ppm. This was further supported by the long range HMBC correlation of 17-COOMe to 17-CO at 168.8 ppm (Supplementary Figure S4). A singlet at 3.75 ppm of 17-COOMe was observed in the proton NMR spectrum for 19,20 *E* vallesamine (Figure 1). In the case of the HSQC NMR spectrum, a correlation between ^1^H and ^13^C suggested carbon of the methyl of 17-COOMe at 53.0 ppm, and HMBC correlation of 17-COOMe to 17-CO at 174.5 confirmed the presence of 19,20 *E* vallesamine (Supplementary Figures S3 and S4).

**Figure 2:**
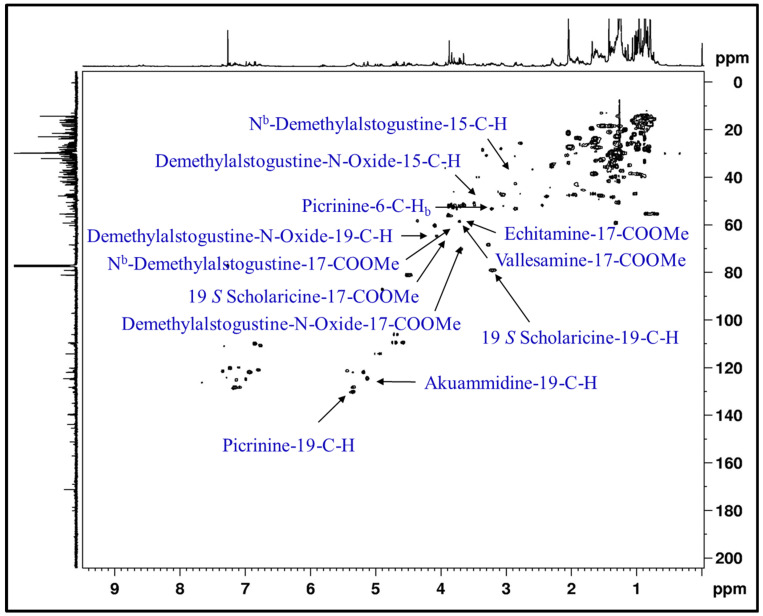
**Identification of HSQC correlation of alkaloids:** Characteristic assignments of alkaloids in 2D ^1^H-^13^C HSQC NMR spectrum of the AS−TB (AS−trunk bark) sample.

The trunk bark of AS contains many indole alkaloids and some of them have been identified and characterized with the help of 1D and 2D NMR experiments. A signal in proton ^1^H NMR at 3.80 ppm (s) indicated the presence of alkaloid Nb-demethylalstogustine N-oxide (Supplementary Figure S5). The HSQC showed a correlation between 3.80 (CH_3_) and 51.3 ppm; and in HMBC, 17-COOMe correlates to 17-CO at 167.2 ppm, indicating the presence of Nb-demethylastogustine N-oxide. The NMR signal at 3.84 ppm in ^1^H NMR spectrum of trunk bark indicated the presence of Nb-demethylalstogustine, which was supported by the presence of COSY correlation between 4.05 (19-H) and 1.85 (18-CH_3_). In 2D HSQC spectrum, a direct bond correlation was observed between 3.84 (CH_3_) and 51.5 ppm; and multiple bond correlations of 17-COOMe to 17-CO at 167.5 ppm further supported the presence of Nb-demethylalstogustine in the trunk bark. Echitamine is one of the most important alkaloids present in the AS and in ^1^H NMR spectrum, it showed a singlet at 3.73 ppm and indicated the presence of echitamine. HSQC showed a correlation between 3.73 (^1^H) and 51.3 (^13^C) and confirmed the presence of echitamine, which was further supported by HMBC correlation between 3.73 and 171.07 ppm (Figure 2 and Supplementary Figures S6 and S7).

The flowers of AS have been also investigated for the alkaloids and several alkaloids have been identified in them, such as picrinine, akuammidine, 19 *S* scholaricine, 19,20 *E* vallesamine and Nb-demethylalstogustine (Supplementary Figures S8–S11). Assignments of alkaloids in the ^1^H NMR spectrum of the stem samples were also performed using the 2D NMR experiments. No signals of picralinal were observed in the trunk barks or in the stem samples of AS (Figure 2).

Based on the previously published literature and experimental NMR data, important ^1^H and ^13^C NMR chemical shift (in ppm) of identified alkaloids is summarized in the Table 1, and the structure of identified alkaloids are shown in Supplementary Figure S12.

### 2.2. Tissue-Specific Profiling of Alkaloids in AS

**Figure 3:**
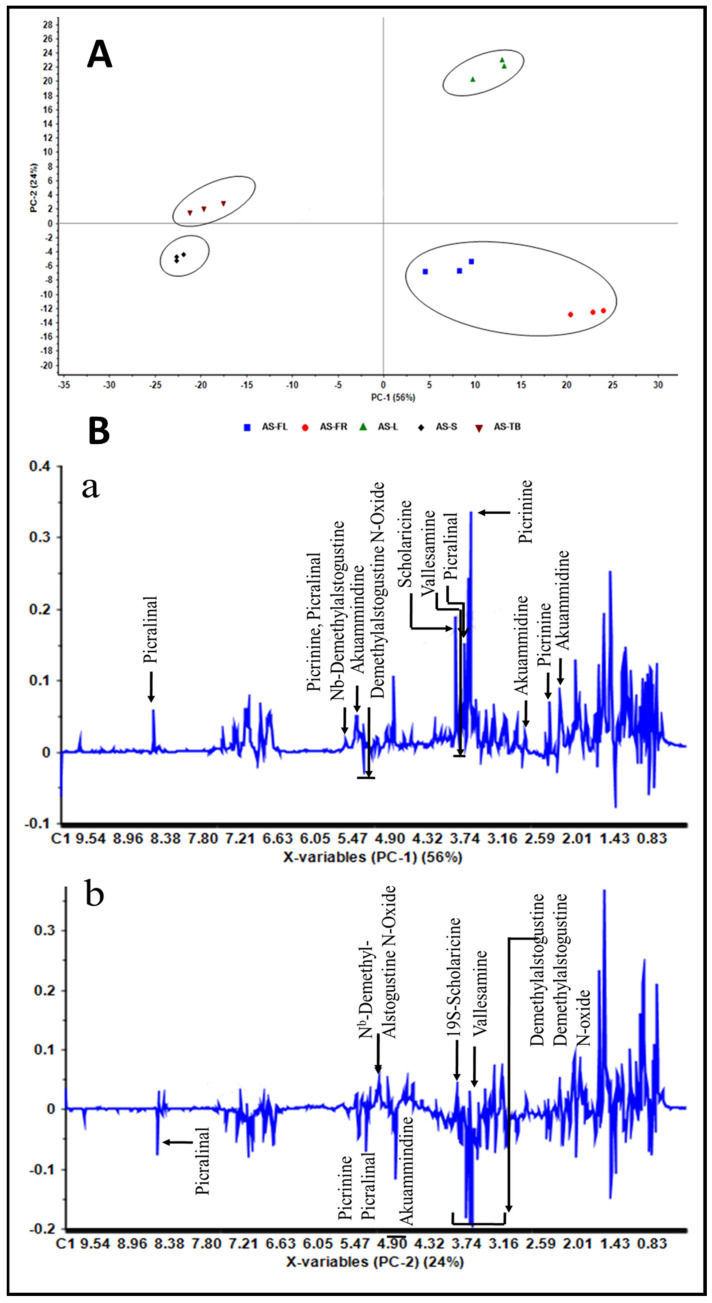
**Principal component analysis of different parts of AS:** (**A**) PCA 2D scatter plots of ^1^H NMR spectra of AS. PCA showed four major group separations: (a) combined group of AS-FL and AS-FR, (b) AS-L, (c) AS-S, and (d) AS-TB. (**B**) The corresponding loading plots for (**a**) principal component 1 (PC-1) and (**b**) principal component 2 (PC-2), showing variability of the alkaloids in PC analysis.

Qualitative analysis of the ^1^H NMR spectra of flowers, fruits, leaves, stems and trunk barks are shown in Supplementary Figure S13. Multivariate statistical analysis such as principal component analysis (PCA) was performed on the ^1^H NMR data of fruits, flowers, stems, leaves and trunk barks of AS. The first two components i.e., PC-1 and PC-2, explained 80% of the total variance and showed the differentiation among the different parts of this plant. PCA showed four major clusters i.e., (a) combined group of flowers and fruits, (b) leaves, (c) stems, and (d) trunk bark (Figure 3).

**Figure 4:**
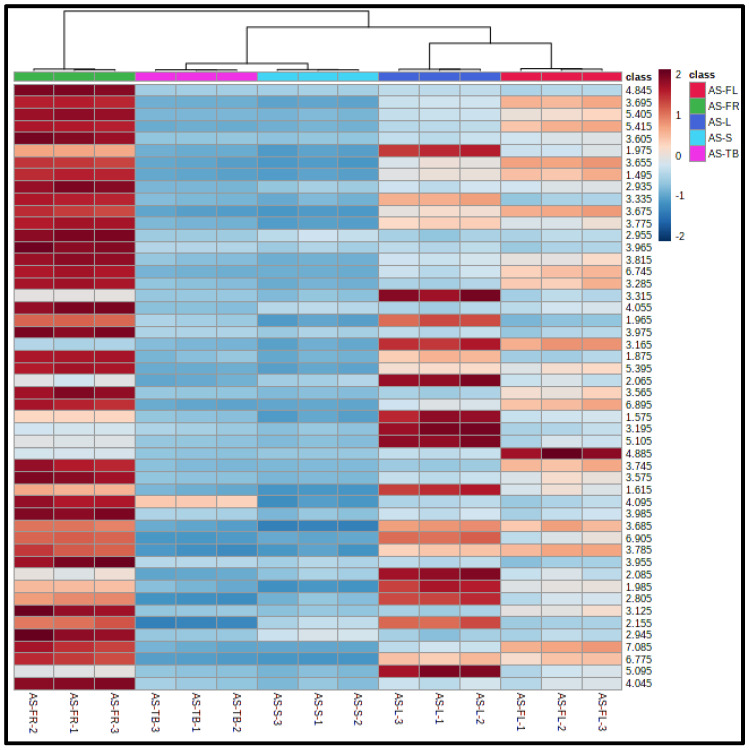
**Hierarchical clustering analysis:** Heatmap demonstrating the differences in the levels of top 50 features in different parts of AS. (Note: The features shown in heatmap are chemical shift values utilized for clustering analysis.).

To demonstrate an overall distribution of metabolites in plant parts and classification of samples, clustering analysis was performed. The results from the clustering analysis were demonstrated in a heatmap using analysis of variance (ANOVA) in Metaboanalyst. The metabolites levels are highest in fruits, followed by leaf samples (Figure 4). The heatmap demonstrated that the levels of metabolites are lowest in stems and trunk barks of AS.

Supervised partial least square discriminant analysis (PLS-DA) was utilized for pattern recognition and facilitated the characterization of discriminant alkaloids in different plant parts. The cross-validation parameters (Q^2^ and R^2^) were derived and found appropriate for deriving a PLS-DA model from ^1^H NMR spectra of the different parts of AS. PLS-DA also showed four group separations: (a) a mixed group of AS-FR and AS-FL, (b) AS-L, (c) AS-S and (d) AS-TB. This PLS-DA model facilitated the cluster formation and sample classification for this plant (Supplementary Figure S14).

The ^1^H NMR-based quantitative analysis demonstrated the highest concentration of picrinine and picralinal in fruits and flowers. The concentration of picrinine and picralinal in fruits were 73.41 ± 0.3292 and 38.13 ± 0.1472, respectively (in µg/g of dry weight of the sample). Echitamine was found to be highest in the AS-TB, which was calculated to be 14.21 ± 1.123 µg/g of dry weight of sample. The concentration of each alkaloid in different parts of the plant is depicted in Table S1. Statistical significance was evaluated using the student’s t-test and shown in Table S2.

**Figure 5:**
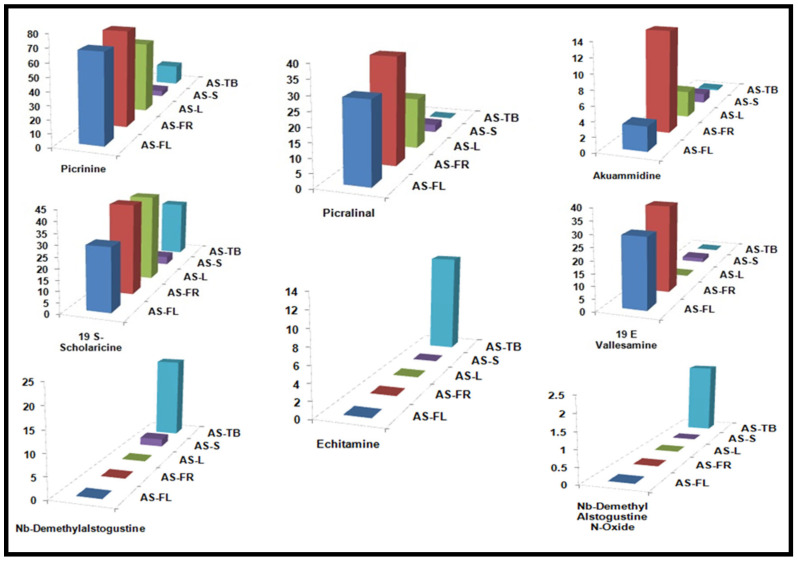
**Quantitative analysis of alkaloids in different plant parts:** Graphical representation of the distribution of alkaloids in the sample obtained from different parts of AS (results expressed as µg/g of dry weight of sample).

A histogram shows the variability among the composition and concentration of alkaloids in the samples obtained from different parts of AS. Graphical representation demonstrates that the concentration of picrinine was lowest in stems and highest in fruits. Picralinal concentration was highest in fruits as well as flowers, whereas it was absent in trunk barks. Akuammidine was found to be low in all of the parts except fruits (Figure 5). Additionally, Echitamine, Nb-demethylalstogustine N-Oxide and Nb-demethylalstogustine were mainly found in the trunk barks. The echitamine was found abundantly only in the trunk bark samples.

### 2.3. Seasonal Variation of Alkaloids in AS

**Figure 6:**
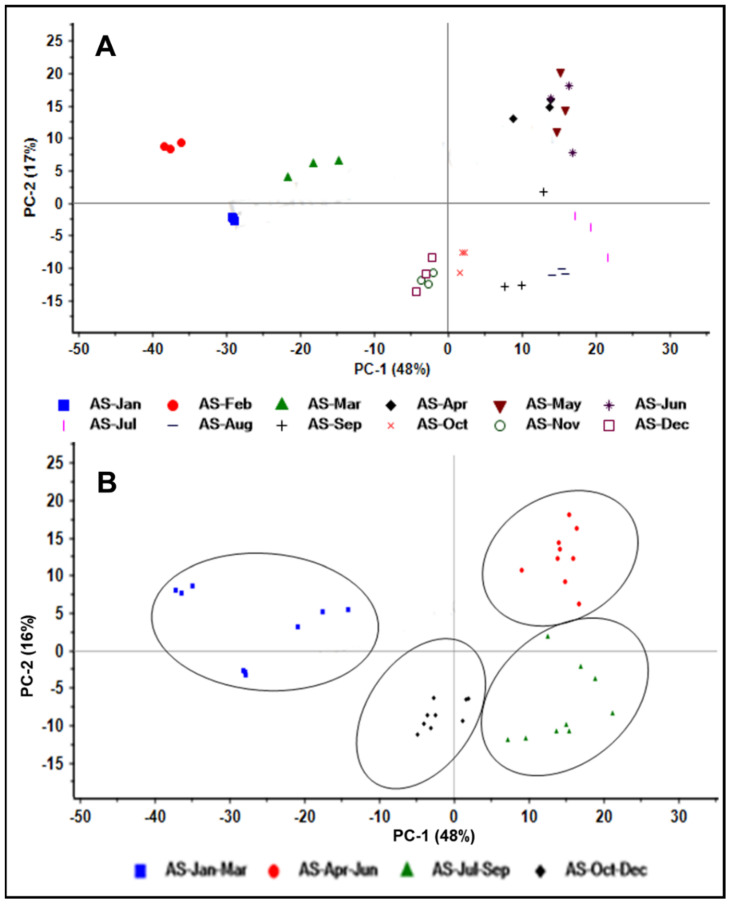
**Principal component analysis of leaf samples of AS from a whole year:** (**A**) 2D scatter PC score plots of ^1^H NMR spectra of AS showing the distribution of monthly collected samples in the scatter scores plot. (**B**) 2D scatter scores plot of ^1^H NMR spectra of AS showed four major groups, i.e., (a) AS−Jan−Mar, (b) AS−Apr−Jun, (c) AS−Jul−Sep and (d) AS−Oct−Dec.

Qualitative analysis of the ^1^H NMR spectra of all months’ samples are shown in Supplementary Figure S15. To investigate the effect of seasonal variation on the production of alkaloids in leaves of this plant, PCA was carried out on ^1^H NMR spectra of leaf samples for a whole year. In total, 64% of the variability was explained by 2D PC scatter scores plot, whereas PC-1 explained 48% of the total variance, and PC-2 explained 16% of the variability (Figure 6). Positive loadings were mainly dominated by 19 *S* scholaricine, and the negative loadings were contributed by the alkaloids such as picrinine, picralinal and akuammidine (Supplementary Figure S16). Semi-quantitative PCA was also supported by the quantitative method and demonstrated that the concentration of picrinine and picralinal were highest in the month group referred to as AS (Jul–Sep) and found to be 181.45 ± 0.9948 µg/g and 127.40 ± 19.06 µg/g, respectively. Similarly, the highest concentration of 19 *S* scholaricine was found in the month group AS (Apr–Jun), which was 178.08 ± 19.88 µg/g of dry weight of the sample. PLS-DA was also performed on the ^1^H NMR data and demonstrated Q^2^ of 0.576 and R^2^ of 0.643 (Supplementary Figure S17).

Average concentration of each identified alkaloid was calculated in groups of three months i.e., (a) AS (Jan–Mar), (b) AS (Apr–Jun), (c) AS (Jul–Sep) and (d) AS (Oct–Dec) and depicted in Table S3. Values of *p* ≤ 0.05 showed the significance of metabolites among four groups of the samples from a whole year. *p* ≤ 0.01 indicated the alkaloids contributed most significantly for the variation among groups (Table S4).

## 3. Discussion

Alkaloids are extremely diverse in terms of chemical structure and biosynthetic pathways [38]. Hence, an analytical approach is much needed to identify the chemical structure and determine the composition of alkaloids in plant. The advancement in NMR as an analytical tool has provided an excellent opportunity to characterize and quantify secondary metabolites in plant extracts [39]. The composition and distribution of alkaloids in leaves, stems, trunk barks, flowers, and fruits of AS needed to be determined before they could be used for any pharmacological purposes. NMR-based metabolomics could play a vital role in providing a clear metabolic picture of different plant parts of AS [40]. Additionally, the identification of alkaloids in plant parts will facilitate an efficient isolation of the alkaloids of interest from the extracts of AS. The NMR results of ethanolic extract in various solvents do not show good sensitivity or resolution for alkaloids (Supplementary Figure S1). As a result, selective alkaloidal extraction was carried out, leading to better sensitivity and high resolution among alkaloids in alkaloidal fractions. The ^1^H NMR spectra of leaves, flowers, fruits, stems, and trunk barks showed the qualitative variation in the chemical composition (Supplementary Figure S13). NMR profile of the alkaloids in AS is quite different and clearly indicated that the biosynthetic metabolic pathway of the alkaloids could be different in each part of AS. Furthermore, 2D NMR analysis was performed to identify the characteristic chemical shifts of alkaloids in each part of AS. The excellent resolution of COSY, HSQC, and HMBC 2D NMR spectra helped to determine the presence of alkaloids in various parts of AS. One of the distinguished metabolites is echitamine, which is identified by NMR in trunk barks only and not in stems, flowers, fruits, or leaves, suggesting specificity of echitamine in trunk barks. By contrast, NMR studies found that the metabolite profile of stems is quite similar to that of the trunk barks of AS.

An ^1^H NMR coupled with multivariate statistics is an excellent approach for the purpose of extracting inherent chemical information and sample classification for different plant parts as well as seasons [41]. Therefore, the ^1^H NMR-based metabolomic approach could be considered to investigate the relationship between secondary metabolites and tissue specificity and seasonal variations. PCA scatter plots derived from ^1^H NMR data showed sample distribution in a mathematical model with excellent separation as well as clustering among plant parts based on their metabolite profiles (Figure 3A). PC-1 loadings showed a trend in the different parts, moving from negative values to positive values, which indicated the cluster separation due to negative loadings of the Nb-demethylalstogustine N-Oxide and Nb-demethylalstogustine, and positive loadings of picrinine, picralinal, 19 *S* scholaricine and akuammidine. Thus, it can be said that semi-quantitative concentration of picrinine, picralinal, 19 *S* scholaricine and akuammidine is higher in flowers, fruits and leaves than in stems and trunk barks of AS. Examination of PC-2 loadings demonstrated that the group separation was because of the positive loadings of Nb-demethylalstogustine N-Oxide, Nb-demethylalstogustine, 19 *S* scholaricine; and of the negative loading of picrinine, picralinal, and 19,20 *E* vallesamine (Figure 3B). Fruits and flowers showed similar profiles and indicated their strong tissue specificity; while trunk barks and stem samples lie in the same space on PC score plots, strongly suggesting the similar metabolic profiling of alkaloids. The score plots strongly suggested that AS leaves are totally different in their alkaloidal profile (Figure 3A). The full cross-validated score plots showed a statistically significant pattern for the samples, and the quality of the supervised mathematical model was explained by the parameters R^2^ and Q^2^ [42]. Q^2^ was found to be lower than R^2^ and demonstrated the quality of a robust model for variation study. High Q^2^, and R^2^ values indicate that the PLS-DA model illustrates better separation among groups and clustering of similar ^1^H NMR profile samples.

The nature and tissue composition of plant parts make them unique for the production of secondary metabolites with different chemical structures [43]. Plant metabolic studies have suggested that the tissue specific distribution and the regulation of enzymatic reaction via specific pathways could impose variation in plant metabolism [44]. The metabolic results in this study indicated that stems and trunk barks produced almost similar metabolites, which can be supported by the fundamental changes from stem to trunks or similar biochemical mechanisms. The flowers eventually develop into fruits after certain biological processes [45] and share common metabolic biosynthetic pathways, which is well evidenced by the metabolic profiling study in AS. This strongly suggests that the flowers and fruits are likely to contain similar types of alkaloids, and the concentration of alkaloids was indeed highest in these parts, further explaining that the flowers and fruits required a good amount of alkaloids as a deterrent to insects. Cook et. al. has observed similar higher concentration of alkaloid in floral parts than vegetative tissues, which is termed as optimal defense theory [46]. The leaves are completely different to all other plant parts, and contain a huge amount of chlorophyll, which might compete against the metabolic biosynthetic pathways of alkaloids: hence leaves contain a smaller number of alkaloids than other parts of AS.

The impact of seasons on the production of secondary metabolites is a key concern in the search for bioactive compounds [47]. The leaves were studied for assessing the effect of seasonal variation on alkaloid production in AS. Due to the complexity of metabolites in the proton NMR spectrum of leaves, fewer alkaloids were identified. Quantitative analysis showed that all samples contain picrinine, picralinal, akuammidine, and 19 *S* scholaricine, but these alkaloids varied in their amount, and this could be due to the variable weather conditions affecting the metabolic biosynthetic pathways. The ^1^H NMR spectra of different monthly samples showed changes in the alkaloidal profile and demonstrated the qualitative seasonal variation in the alkaloids (Supplementary Figure S15). The PCA score plots for each month samples demonstrated an excellent distribution of samples and showed a characteristic pattern. The samples were combined in groups of 3 months and derived 2D score plots, which reflected the pattern of alkaloid distribution seasonally. The PLS-DA analysis showed the R^2^ value to be higher than 50%, which suggested that the model is quite robust for explaining the distribution as well as the clustering among the leaf samples of this plant collected in each month for a year.

## 4. Materials and Methods

### 4.1. Sample Collection

Various samples like leaves, flowers, fruits, stems, and trunk bark of AS were collected from the CSIR-CDRI campus Lucknow (Lucknow, India). The leaves had been collected in each month for one year (in 2014) to study the seasonal variation. Collected leaf samples were air-dried and kept in air-tight containers. Once samples were collected for 12 months they were utilized for further analysis. The plant was authenticated by an angiosperm taxonomist of CSIR-CDRI and its herbarium specimen, bearing voucher specimen number 24729, has been deposited in our Medicinal Plant Herbarium (Acronym CDRI).

### 4.2. Experimental Design

Leaves, fruits, flowers, stems, and trunk bark (25 g of each) samples were used in triplicate (*n* = 3) for tissue-specific distribution. Triplicate samples of leaves from each month were utilized for studying seasonal variation. All samples were extracted for alkaloids and subjected to NMR analysis followed by quantitative and multivariate statistical analysis.

### 4.3. Metabolite Extraction

Dried ground leaves, fruits, flowers, stems, and trunk bark (25 g of each) were used to measure the variation of alkaloids in different parts of the AS. Air-dried ground leaves (25 g of each) were taken to study the seasonal variation of alkaloids in leaf samples of this plant. All leaves, fruits, flowers, stems, and trunk bark samples were extracted with 95% ethanol (3 × 200 mL), filtered, and concentrated on the rotary evaporator [48]. The ethanolic extract of each of the plant part samples was dissolved in 7% HCl and partitioned with ethyl acetate, resulting in an ethyl acetate fraction-I (AS-EtOAc-I) which was greenish in color and have might contained chlorophyll and other non-polar compounds. HCl was used to convert basic functional groups of alkaloids into hydrochloric acid salts, which could be selectively extracted in water layers (acidic solution). The acidic solution was subsequently basified with liquid ammonia up to pH 9–10 [49]. This was again partitioned with ethyl acetate and finally gave an ethyl acetate fraction-II (AS-EtOAc-II) and water fraction (AS-W). Selective extraction of alkaloids was achieved from the ethanolic extract in the ethyl acetate fraction-II (AS-EtOAc-II).

### 4.4. NMR Analysis

The ^1^H NMR spectra of the ethanolic extract, ethyl acetate-I (AS-EtOAc-I), water (AS-W) and ethyl acetate-II (AS-EtOAc-II) fractions were recorded on Bruker Avance III HD 500 MHz NMR spectrometer equipped with a triple resonance inverse (TXI) probe. Twenty-five mg of each sample of the alkaloidal fraction, i.e., ethyl acetate fraction-II (AS-EtOAc-II) was dissolved in CDCl_3_ (500 μL) and transferred into a 5 mm NMR tube. The characterization of indole alkaloids was done using the 1D and 2D NMR data [29]. Correlation Spectroscopy (COSY), ^1^H-^13^C Heteronuclear Single Quantum Correlation (HSQC) and Heteronuclear Multiple Bond Correlation (HMBC) [36] experiments were used from the Bruker’s library of the experiments.

### 4.5. Quantitative Analysis

The ^1^H NMR-based quantitative analysis of metabolites was done as reported previously [50]. A ^1^H NMR spectrum of gravimetrically weighed (1 mg/mL) pure picrinine was recorded, using the identical conditions as the experimental samples. An artificial signal was added in each spectrum and concentration of alkaloids was determined from the integral areas of ^1^H NMR signal corresponding to that of the artificial signal as reported previously [51].

### 4.6. Multivariate Analysis (Data Reduction and Pattern Recognition)

The ^1^H NMR spectra of all of the samples with a spectral range from 0.69 to 9.50 ppm were taken for binning the data to produce a series of sequentially equal integrated bins of 0.01 ppm width. Regions 1.55–1.60 and 7.25–7.27 ppm were excluded during the binning, which corresponds to residual water signal and residual non-deuterated signal of chloroform, respectively. Each spectrum was binned in 887 equal segments with the integration mode of the sum of intensities. Intensities were scaled to the biggest bucket. Binning was performed using the Bruker AMIX software (version 3.5.5. Bruker Biospin, Germany) [52]. The resulting data matrices were further imported to ‘The Unscrambler X’ Software package (Version 10.0.3, Camo USA, Norway) for multivariate PCA and PLS-DA [51]. Heatmap was generated, using https://www.metaboanalyst.ca/ (accessed on 1 May 2022) software [53]. It was concluded that NMR coupled with PCA and PLS-DA [54] could play an important role in targeted profiling of the alkaloids in different parts of this plant. 

## 5. Conclusions

The phytochemical investigations of alkaloids was carried out using the ^1^H NMR-based metabolic profiling in leaves, stems, trunk barks, flowers, and fruits of AS. NMR, sample classification procedures, and quantitative analysis provided strong evidence for the existence of metabolic profile differentiation among leaves, fruits, flowers, stems, and trunk barks of AS. Chemical shift assignments of alkaloids were carried out using 1D and 2D NMR experiments in the alkaloidal extracts of AS. NMR analysis helped to identify 7 alkaloids in trunk barks, 6 in stems, 5 in flowers as wells as fruits, and 4 alkaloids in leaves of AS. PCA and PLS-DA methods were applied on the ^1^H NMR data and demonstrated that the maximum variation could be achieved in different parts of the plant. Samples collected on a monthly basis for the study of seasonal variation also showed a significant trend in alkaloidal pattern in PCA and PLS-DA. Finally, this work demonstrated that the NMR technique in conjunction with multivariate analysis could play a highly significant role in quality control and herbal extract analysis.

## Figures and Tables

**Table 1:** Characteristic ^1^H and ^13^C NMR chemical shift (ppm) values of the identified alkaloids in the different parts of AS.

S. No.	Compounds	^1^H Chemical Shift (ppm)	^13^C Chemical Shift (ppm)
1	Picrinine	7.13 (d, J = 8.1 Hz), 7.07 (t, J = 8.0 Hz), 6.85 (d, J = 8.1 Hz), 6.78 (t, J = 8.0 Hz), 5.40 (q), 4.85 (m), 3.65 (s), 3.41 (m), 3.30 (m), 2.44 (d, J = 3.1 Hz), 2.24 (m), 2.13 (m), 1.50, 1.85 (m)	172.4, 127, 125, 121.0, 120, 110, 87.3, 51.7, 51.4, 51.3, 40.6, 31.1, 26.1, 12.7
2	Picralinal	8.56 (s), 7.32 (d, J = 7.5 Hz), 7.11 (t, J = 7.4 Hz), 6.88 (t, J = 7.4 Hz), 6.70 (d, J = 7.5 Hz), 5.40 (q), 3.80 (m), 3.69 (s), 3.67 (brs), 3.54 (m), 3.47 (m), 3.11 (m), 2.24 (m), 1.98 (m), 1.50 (m)	197.7, 168.0, 128.7, 126.l, 121.5, 121.0, 110.7, 65.1, 53.6, 52.5, 46.4, 43.1, 31.4, 22.6, 12.8
3	Akuammidine	7.53 (d, J = 7.6 Hz), 7.11 (d, J = 7.6 Hz), 7.04 (t, J = 7.5 Hz), 6.78 (t, J = 7.5 Hz), 5.38 (brq), 3.84 (m), 3.68 (m), 2.94 (s), 2.66 (m), 2.29 (m), 1.86 (m), 1.66 (d, J = 5.0 Hz)	173.8, 121.5, 119.4, 118.1, 116.7, 110.9, 65.7, 51.3, 51.0, 39.5, 29.2, 13.0
4	19 *S* Scholaricine	8.62 (brs), 7.48 (d, J = 7.6 Hz), 7.35 (d, J = 7.6 Hz), 7.04 (t, J = 7.6 Hz), 3.88 (s), 3.28 (m), 3.1 (m), 2.02 (m), 1.72 (m), 1.17 (m)	168.8, 141.2, 122.5, 119.4, 118.1, 96.8, 68.5, 52.1, 31.0, 29.4, 12.5
5	19,20 *E* Vallesamine	7.67 (d, J = 7.6 Hz), 7.30 (d, J = 7.6 Hz), 7.17 (t, J = 7.6 Hz), 7.07 (t, J = 7.6 Hz), 5.55 (q), 4.84 (m), 4.21 (d, J = 10.8 Hz), 3.75 (s), 3.63 (m), 2.33 (m), 1.90 (m), 1.74 (m)	174.5, 123.0, 122.5, 119.4, 118.1, 110.0, 70.4, 58.5, 53.0, 36.2, 23.9, 14.1
6	Nb-Demethylalstogustine N-Oxide	7.67 (d, J = 7.5 Hz), 7.41 (t, J = 7.5 Hz), 6.97 (t, J = 7.5 Hz), 6.86 (d, J = 7.5 Hz), 4.18 (q), 3.80 (s), 3.44 (m), 3.20 (m), 1.38 (m), 1.29 (m)	167.2, 129.1, 121.5, 120.1, 110.0, 103.7, 68.6, 51.3, 24.8, 24.0, 20.3
7	Nb-Demethylalstogustine	7.25 (d, J = 7.6 Hz), 7.18 (t, J = 7.6 Hz), 6.93 (t, J = 7.6 Hz), 6.86 (d, J = 7.6 Hz), 4.05, 3.84 (s), 3.09 (m), 2.35 (m), 1.85, 1.49 (m), 1.24 (m)	167.5, 129.1, 122.5, 121.1, 110.2, 103.1, 68.0, 51.5, 44.4, 28.5, 26.1, 12.7
8	Echitamine	7.74 (d, J = 7.5 Hz), 7.72 (d, J = 7.5 Hz), 7.40 (t, J = 7.5 Hz), 6.25 (t, J = 7.5 Hz), 5.73 (q), 3.73 (s), 3.29 (s), 3.16 (m), 2.24 (m), 2.02 (m)	171.0, 157.3, 129.8, 128.7, 126.7, 119.5, 110.6, 66.3, 64.5, 51.8, 29.2

## Data Availability

The data acquired for this study is included in this manuscript and associated supplementary materials.

## Supplementary Materials

The supporting information can be downloaded at: https://www.mdpi.com/article/10.3390/metabo12070607/s1. Figure S1: Comparison of the sensitivity of alkaloids detection.; Figure S2: Selective extraction of the alkaloids.; Figure S3: 2D HSQC of fruit sample.; Figure S4: 2D HMBC of fruit sample.; Figure S5: 1H NMR of trunk bark sample.; Figure S6: 2D COSY NMR spectrum of trunk bark.; Figure S7: HMBC of trunk bark.; Figure S8a: 1H NMR of flower sample.; Figure S8b: 1H NMR of flower sample.; Figure S8c: 1H NMR of flower sample.; Figure S9: 2D COSY NMR of flower sample.; Figure S10: HSQC of flower sample.; Figure S11: HMBC of flower sample.; Figure S12: Chemical structure of alkaloids.; Figure S13: 1H NMR data comparison of different parts of plant. ; Figure S14: PLS-DA analysis.; Figure S15: Comparative 1H NMR spectra of seasonal variation.; Figure S16: Loading derived from PCA of seasonal variation data.; Figure S17: PLS DA of seasonal variation in group of 4 months., Table S1: Concentration of alkaloids in plant parts.; Table S2: Significant difference between plant parts.; Table S3: Average concentration of alkaloids in group of 4 months. Table S4: Significant difference in concentration between group of 4 months.
