# Supplementary material for: Assessment of Tissue Specific Distribution and Seasonal Variation of Alkaloids in Alstonia scholaris
Source: Metabolites. 2022 Jun 30;12(7):607. doi: 10.3390/metabo12070607 (PMC9319449; doi:10.3390/metabo12070607)
Supplement: Supplementary file 1 [file metabolites-12-00607-s001.zip › metabolites-1781445-supplementary.pdf]

*Supplementary Materials*

# Assessment of tissue specific distribution and seasonal variation of alkaloids in *Alstonia scholaris*

Rohit Mahar[1], Nagarajan Manivel[1], Sanjeev Kanojiya[1], Dipak K. Mishra[1], Sanjeev K. Shukla[1,*]

[1] Sophisticated Analytical Instrument Facility and Research, CSIR-Central Drug Research Institute, Lucknow-226031, INDIA; rohitmahar4u@gmail.com (R.M.); nagambt@gmail.com (N.M.); sanjeev_kanojiya@cdri.res.in (S.K.); dk_mishra@cdri.res.in (D.K.M.)

* Correspondence: skshukla@cdri.res.in, sanshukla@gmail.com (S.K.S.)

**Figures**

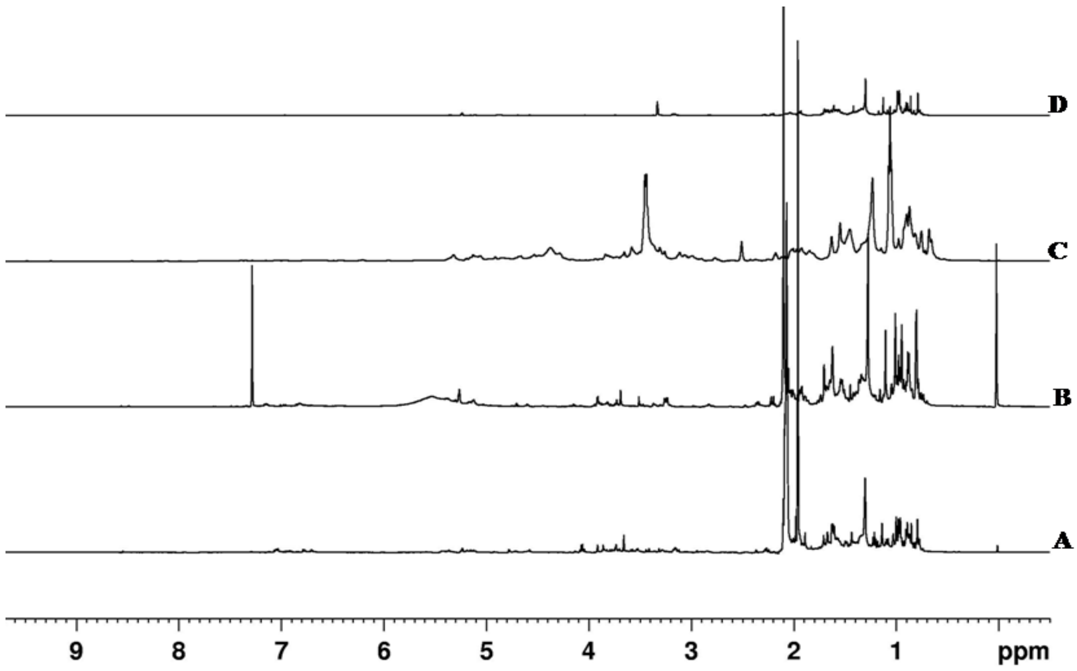


**Supplementary Figure S1: Comparison of the sensitivity of alkaloids detection.** Stacked plot of $^1$H NMR spectra of ethanolic extract of the leaves sample of *A. scholaris* in (A) Acetone-$d_6$, (B) $CDCl_3$, (C) MeOD-$d_4$ and (D) DMSO-$d_6$

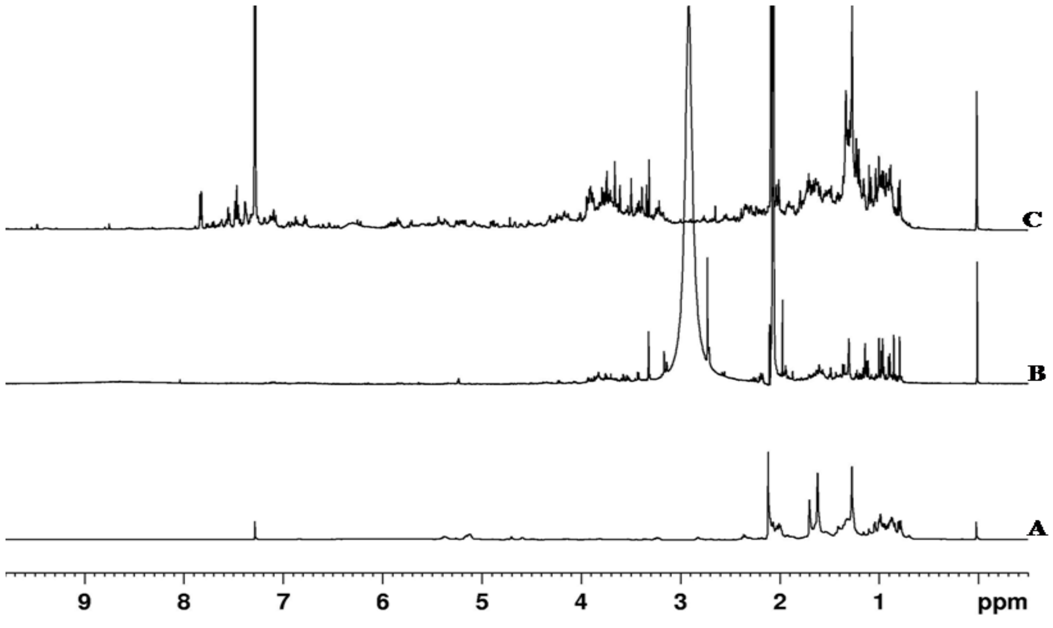


**Supplementary Figure S2: Selective extraction of the alkaloids.** Stacked plot of the $^1$H NMR spectra of fractions of ethanolic extract of the leaves of *A. scholaris*, (A) ethyl Acetate fraction-I (AS-EtOAc-I), (B) Water fraction (AS-W) and (C) ethyl acetate fraction-II (AS-EtOAc-II)

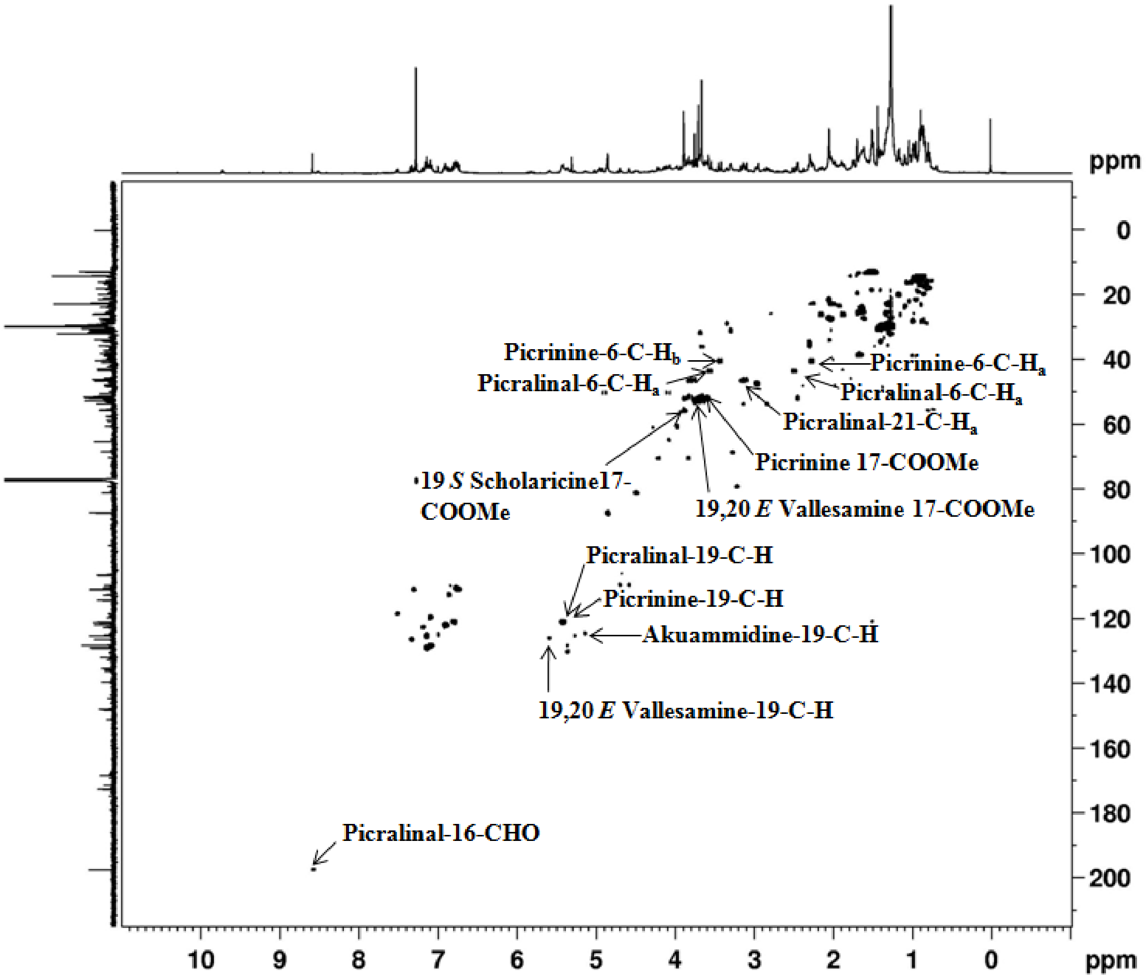


**Supplementary Figure S3: 2D HSQC of fruit sample.** Assignments of alkaloids in the $^{1}$H-$^{13}$C HSQC NMR spectrum of AS-FR (*A. scholaris*-fruits) sample. Characteristic correlations have been shown for the alkaloids

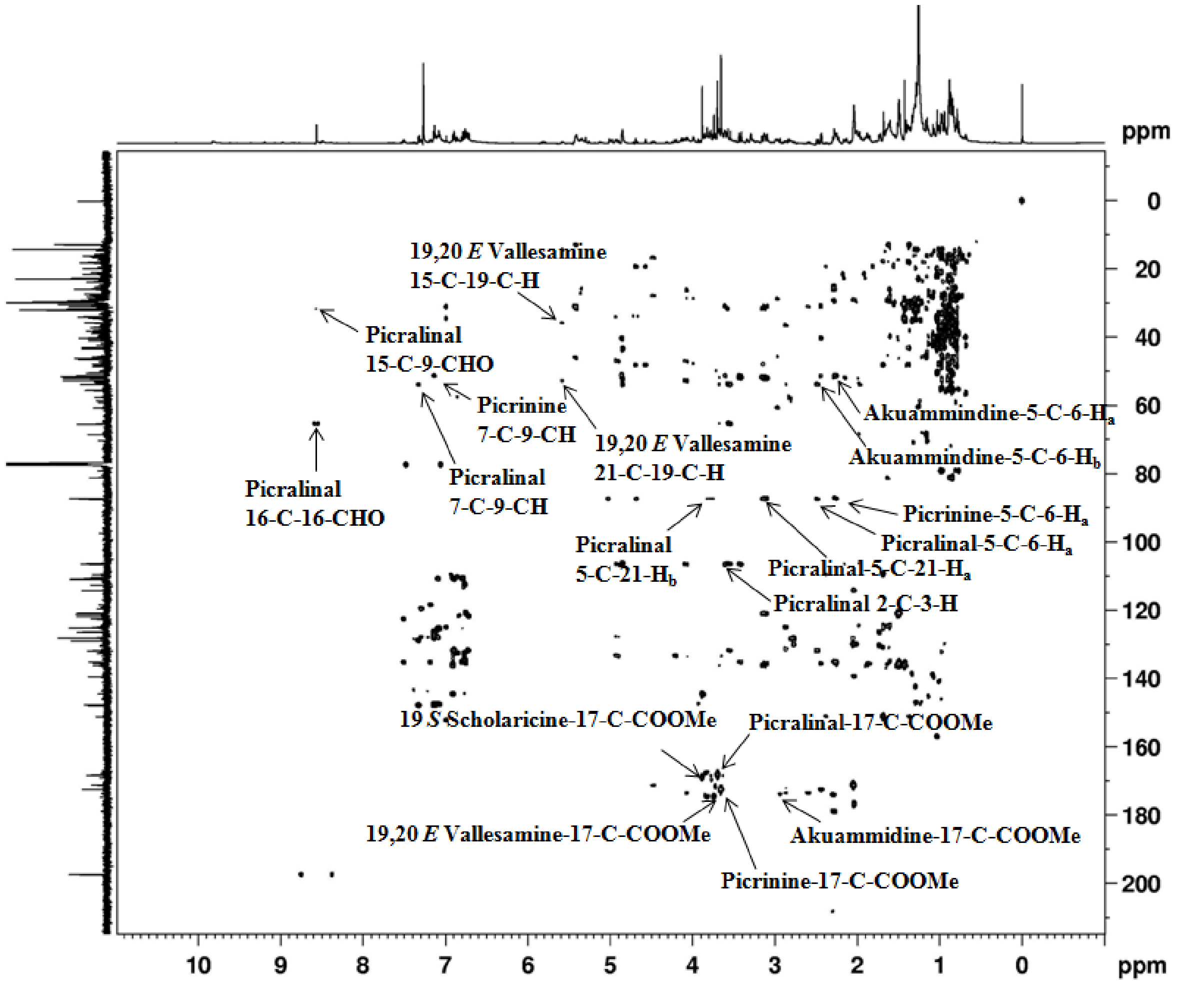


**Supplementary Figure S4: 2D HMBC of fruit sample.** Assignments of alkaloids in 2D $^{1}H$-$^{13}C$ HMBC NMR spectrum of AS-FR (*A. scholaris*-fruits) sample

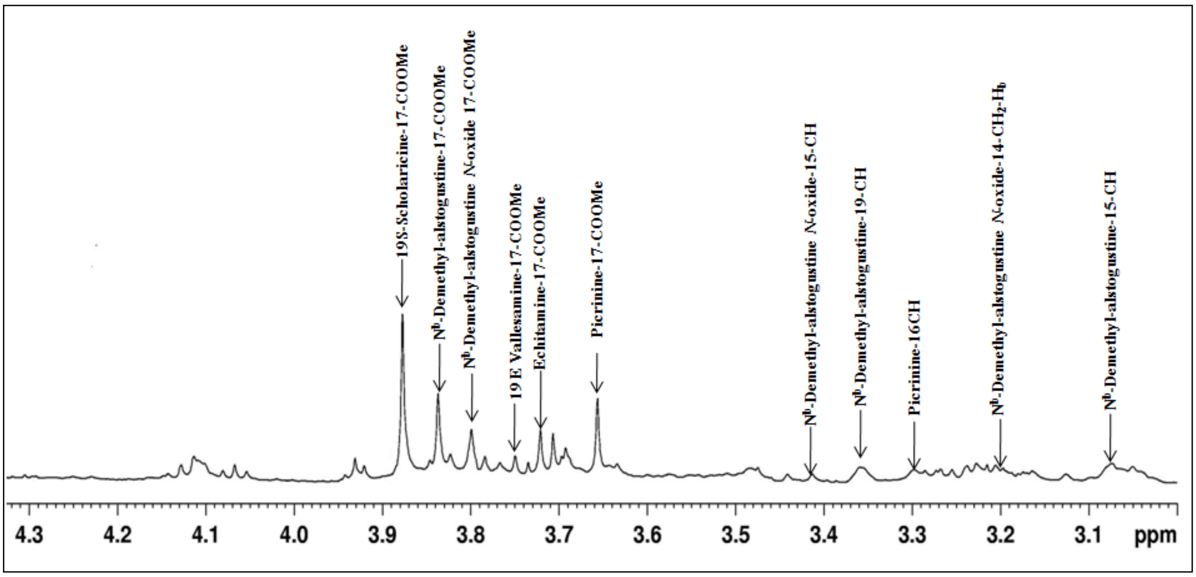

**Supplementary Figure S5: $^{1}$H NMR of trunk bark sample.** Representative $^{1}$H NMR spectrum showing the assignments of alkaloids in (expanded region 2.70 ppm to 4.30 ppm) of AS-TB (*A. scholaris* trunk bark) sample

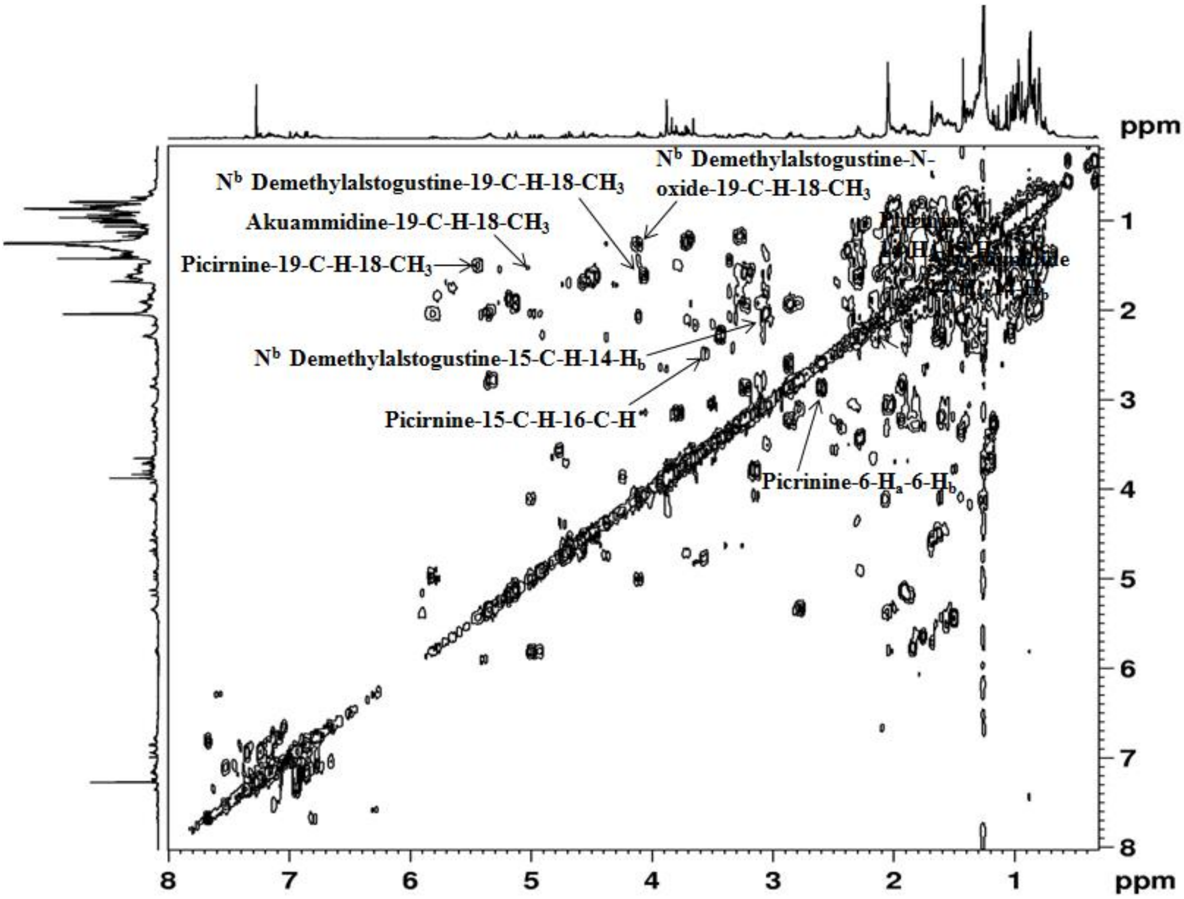


**Supplementary Figure S6: 2D COSY NMR spectrum of trunk bark.** Characteristics assignments of alkaloids in 2D $^{1}$H-$^{1}$H COSY NMR spectrum of the AS-TB (*A. scholaris*-trunk bark) sample

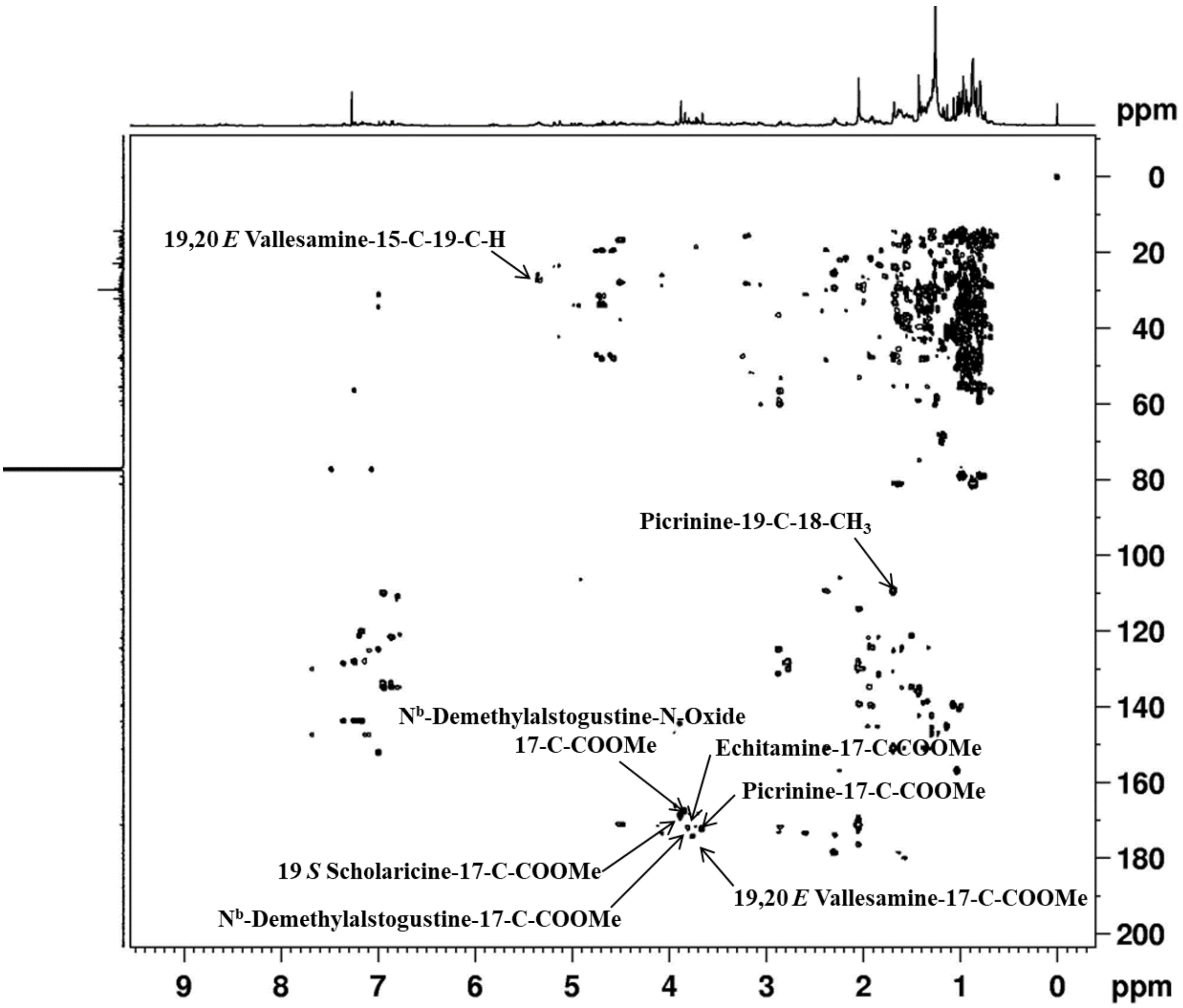


**Supplementary Figure S7: HMBC of trunk bark.** Characteristics correlations of alkaloids in 2D $^{1}H$-$^{13}C$ HMBC NMR spectrum of AS-TB (*A. scholaris*-trunk bark) sample

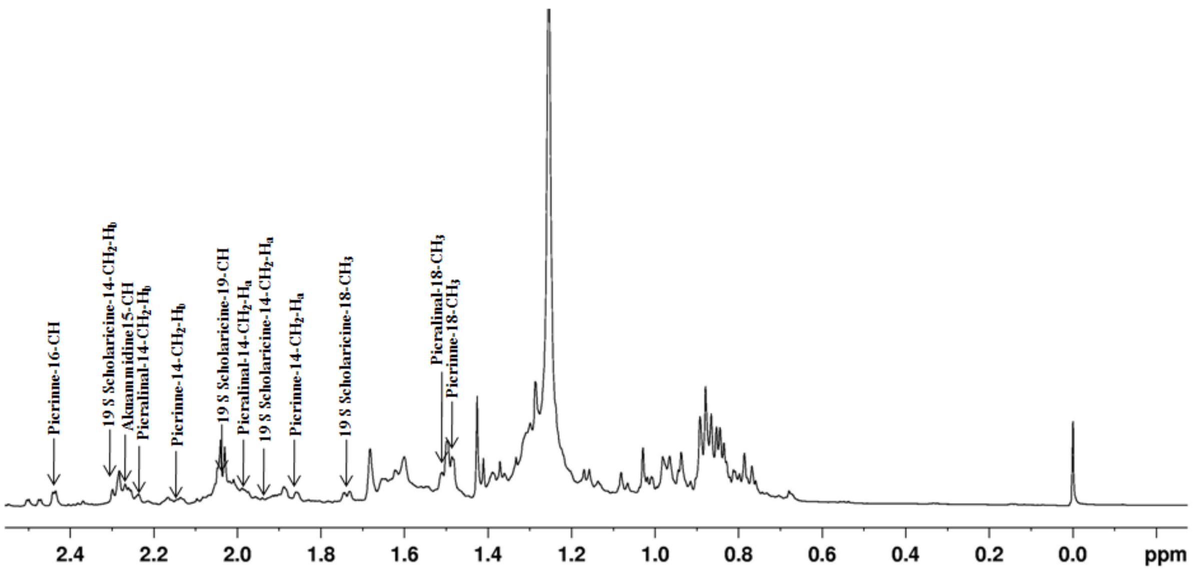

**Supplementary Figure S8a: $^1$H NMR of flower sample.** Assignments of alkaloids in $^1$H NMR spectrum (expanded region from -0.50 ppm to 2.50 ppm) of AS-FL (*A. scholaris*-flowers) sample

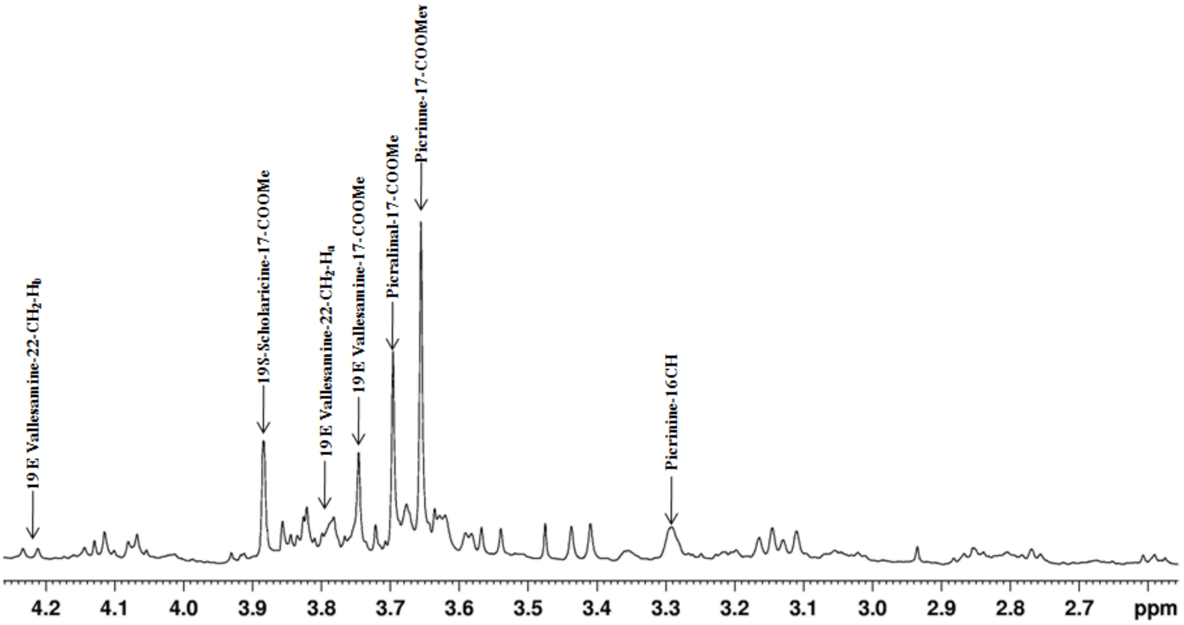


**Supplementary Figure S8b: $^1$H NMR of flower sample.** Assignments of alkaloids in $^1$H NMR spectrum (expanded region 2.50 ppm to 4.30 ppm) of AS-FL (*A. scholaris*-flowers) sample

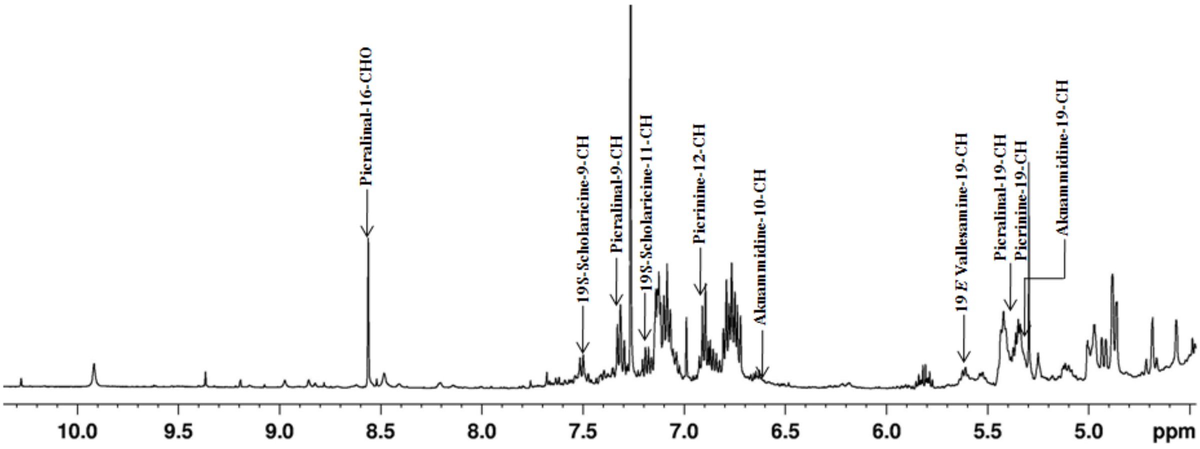


**Supplementary Figure S8c: $^1$H NMR of flower sample.** Assignments of alkaloids in $^1$H NMR spectrum (expanded region 4.00 ppm to 9.50 ppm) of AS-FL (*A. scholaris*-flowers) sample

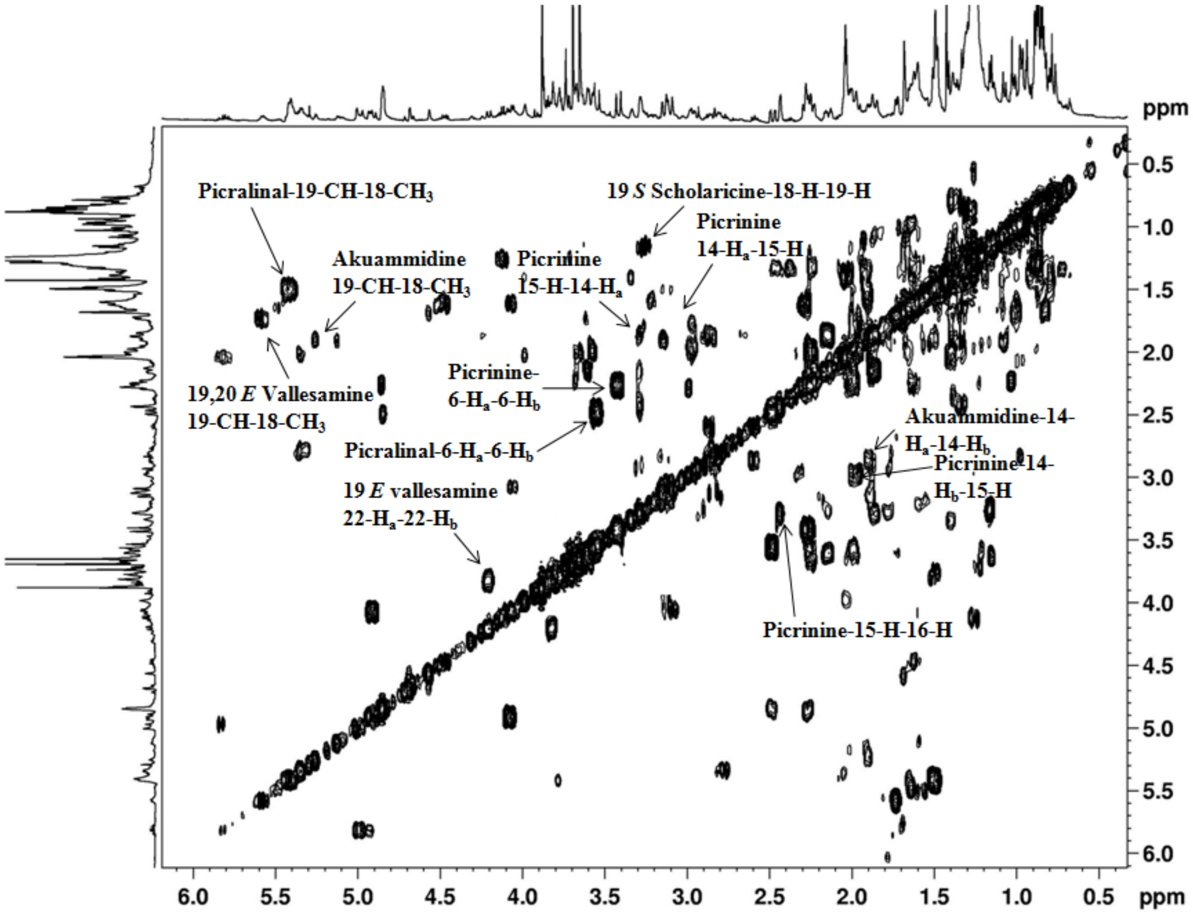


**Supplementary Figure S9: 2D COSY NMR of flower sample.** Assignments of alkaloids in $^{1}H$-$^{1}H$ COSY NMR spectrum of AS-FL (*A. scholaris*-flowers) sample

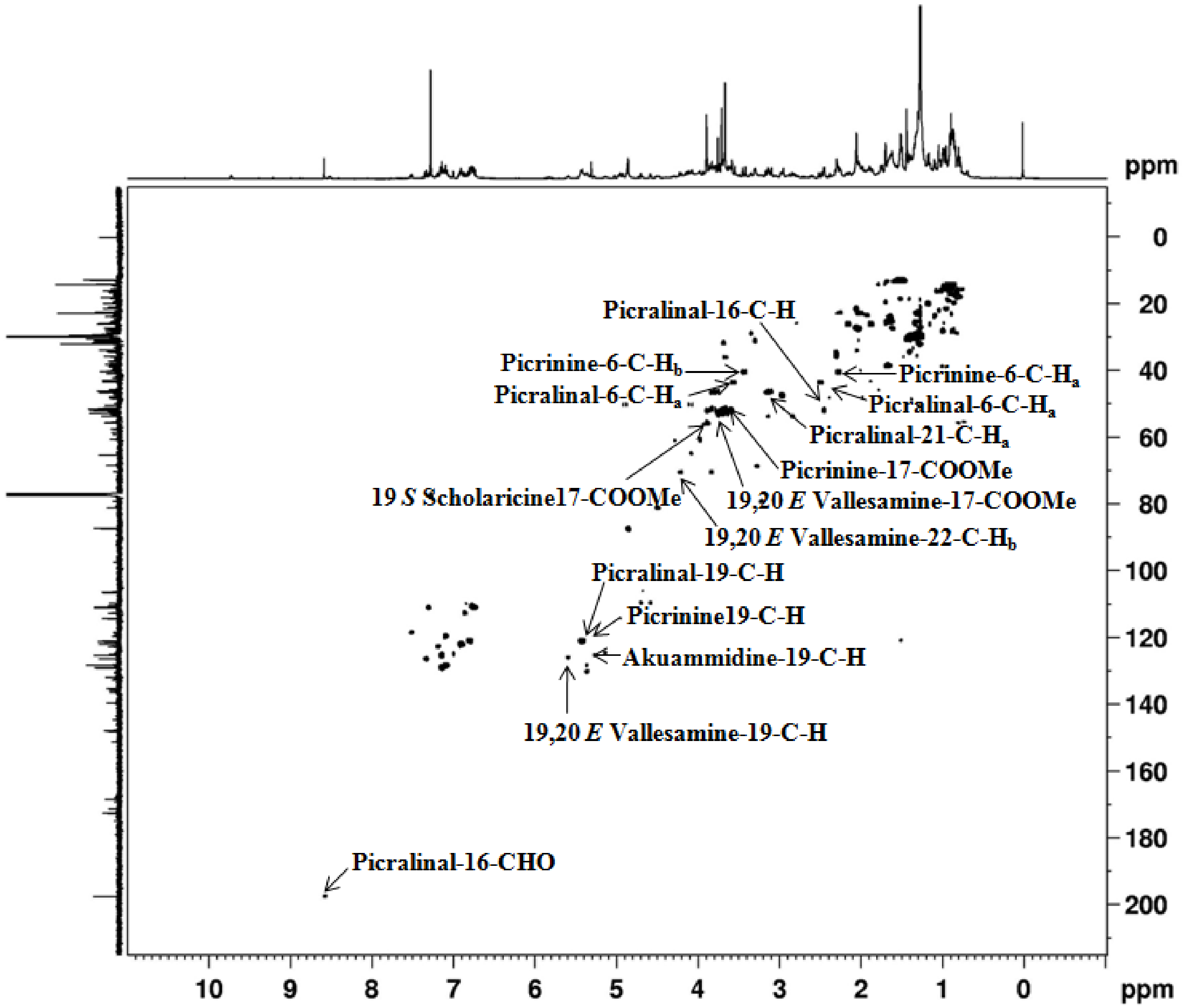


**Supplementary Figure S10: HSQC of flower sample.** Characteristics assignments of alkaloids in the $^{1}H$-$^{13}C$ HSQC NMR spectrum of AS-FL (*A. scholaris*-flowers) sample

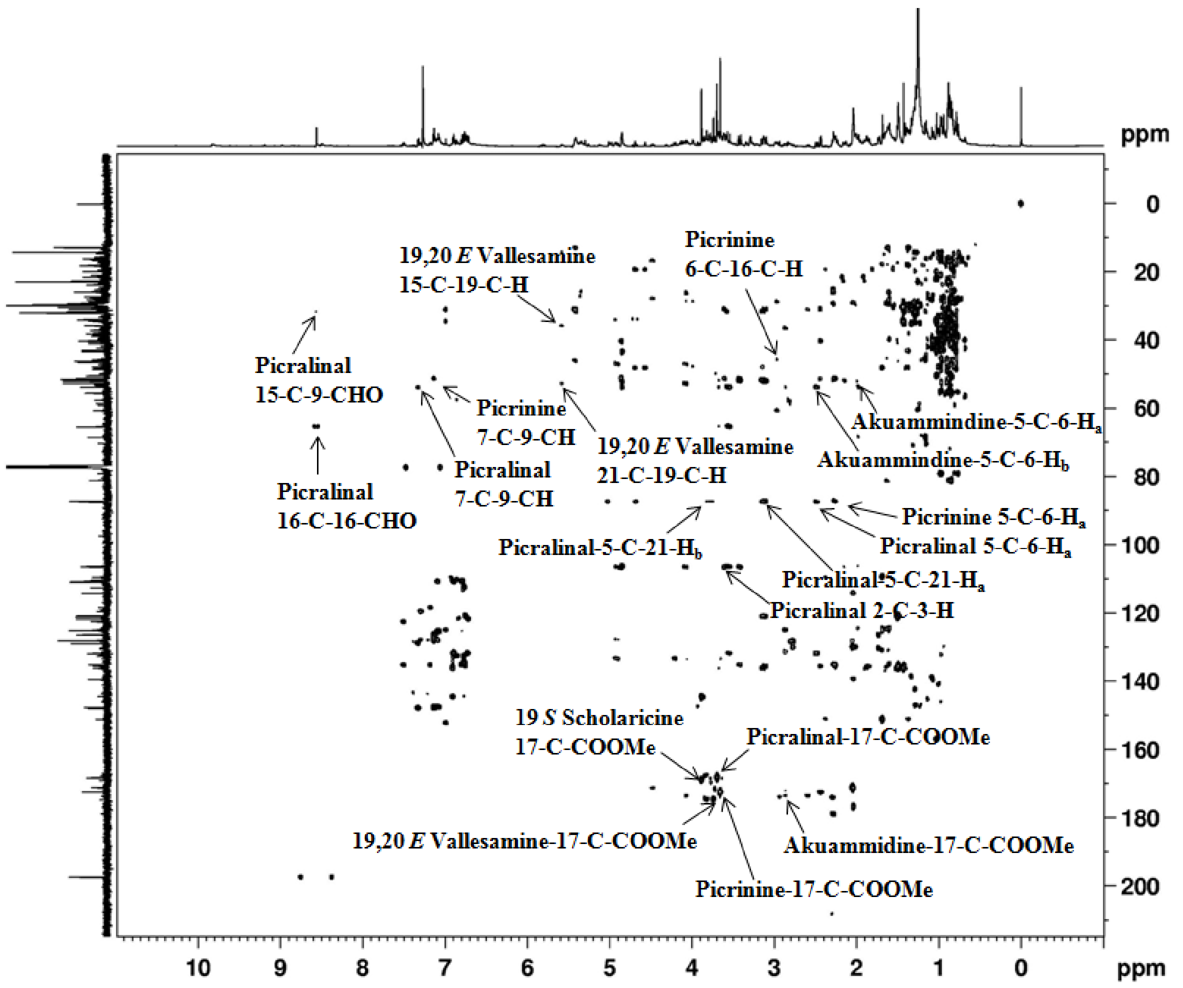


**Supplementary Figure S11: HMBC of flower sample.** Assignments of alkaloids in 2D $^{1}H$-$^{13}C$ HMBC NMR spectrum of AS-FL (*A. scholaris*-flowers) sample

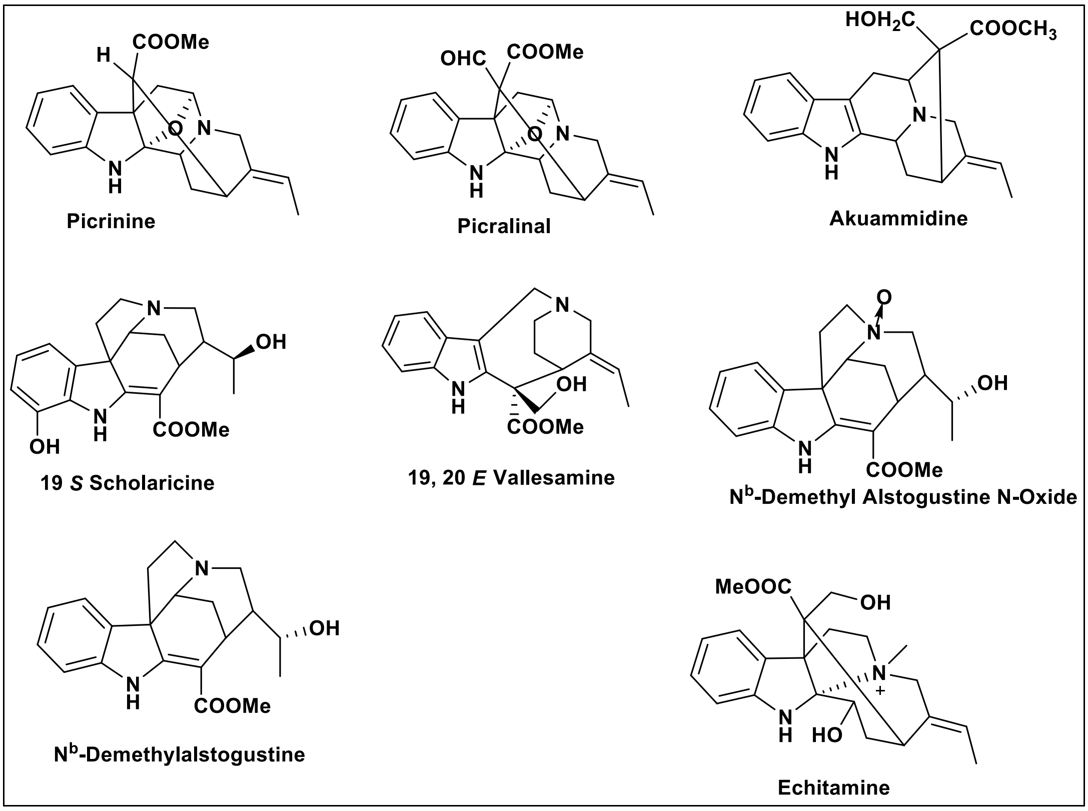


**Supplementary Figure S12: Chemical structure of alkaloids.** Structures of identified alkaloids in different parts of *A. Scholaris*

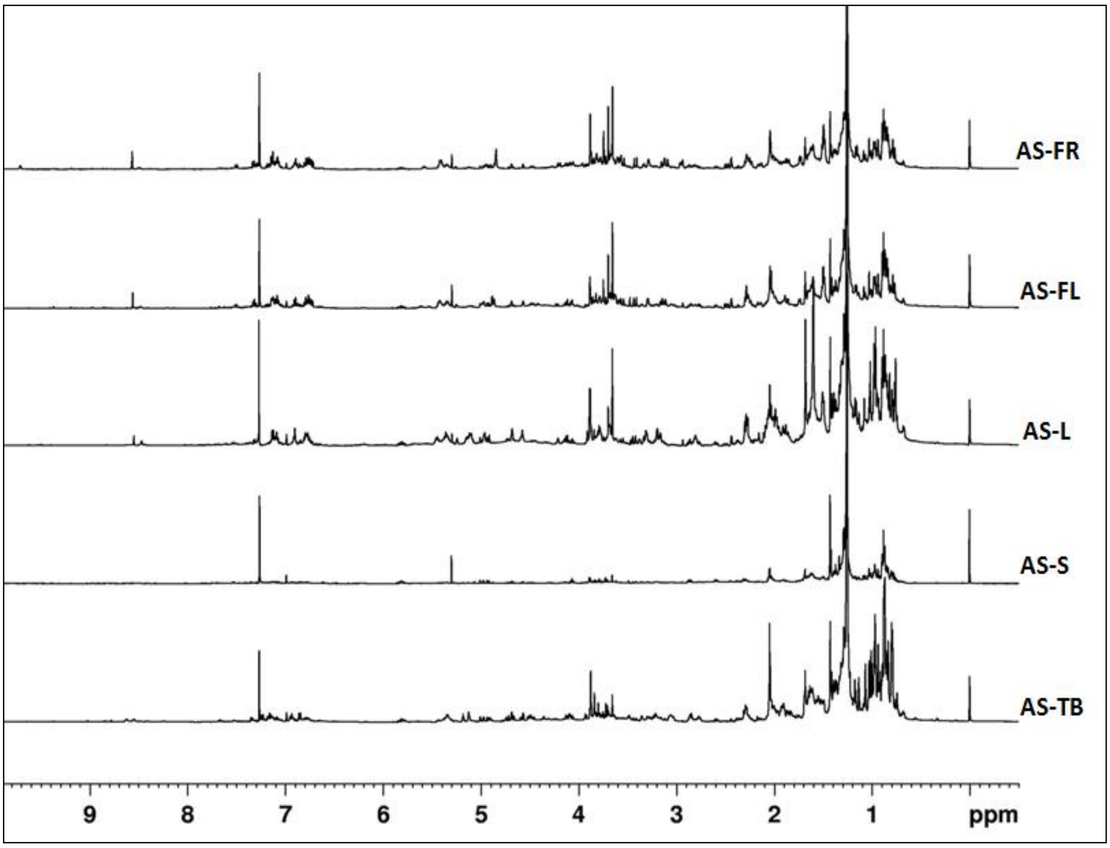


**Supplementary Figure S13: $^{1}$H NMR data comparison of different parts of plant.** Stacked plot of the $^{1}$H NMR spectra showing the comparison of (a) *A. scholaris*-trunk bark (AS-TB), (b) *A. scholaris*-stems (AS-S), (c) *A. scholaris*-leaves (AS-L), (d) *A. scholaris*-flowers (AS-FL) and (e) *A. scholaris*-fruits (AS-FR)

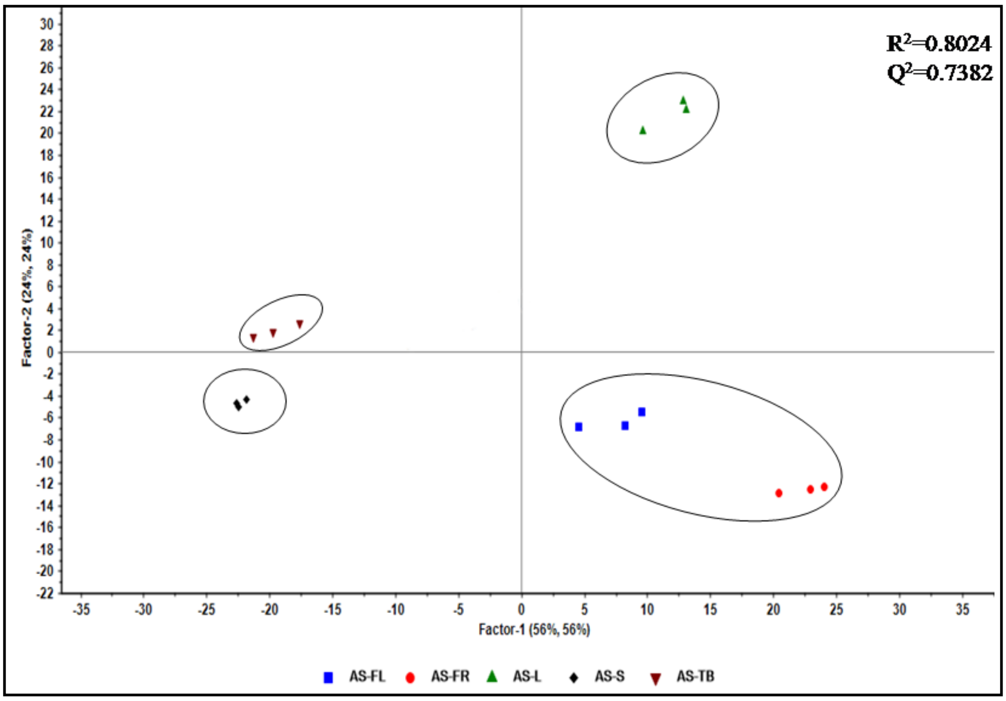


**Supplementary Figure S14: PLS-DA analysis.** PLS-DA (2D scatter scores plot) of $^{1}$H NMR data of *A. scholaris*, showed four major group separation (a) Intermixing groups of AS-FL and AS-FR, (b) AS-L, (c) AS-S and AS-TB, with this it showed the $Q^2$ predictive ability with $R^2$ statistical validity

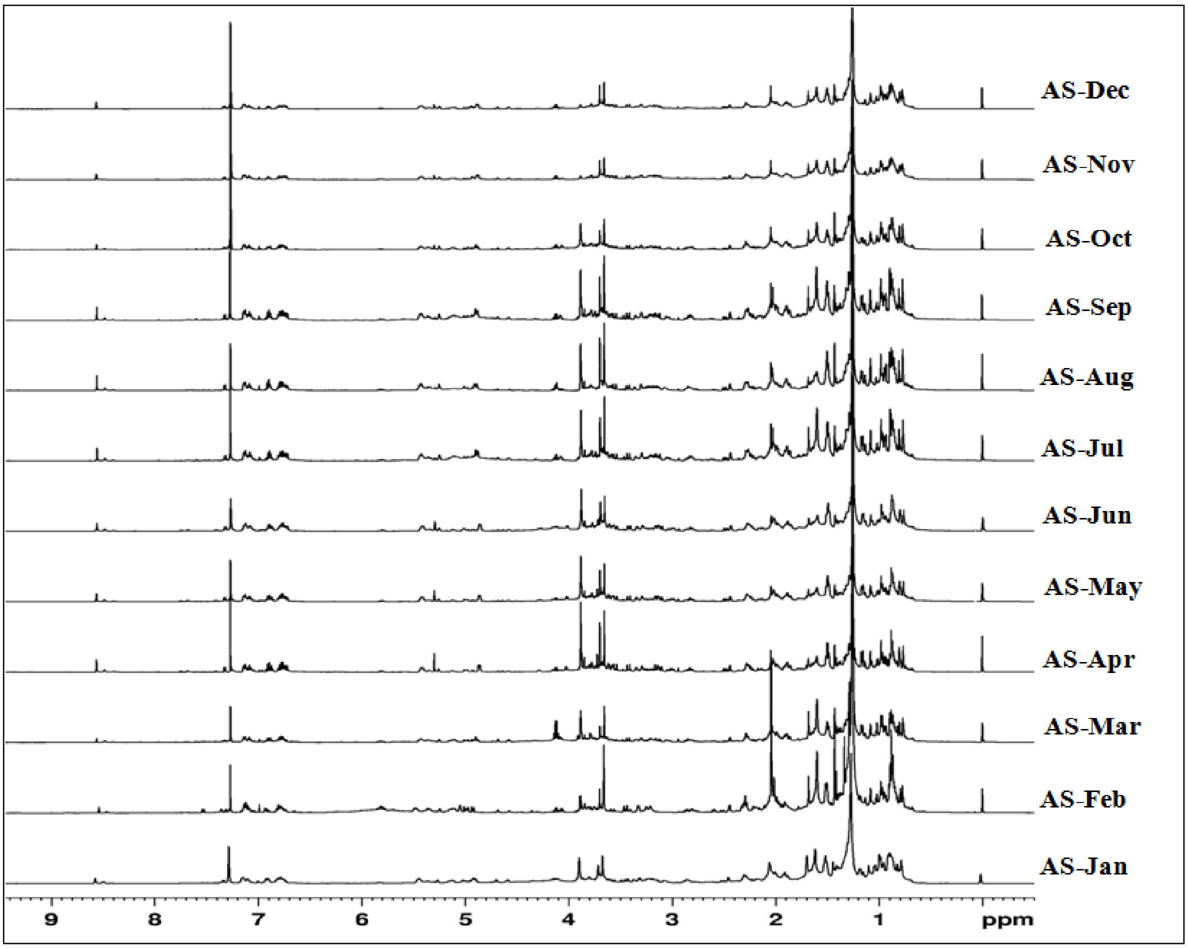


**Supplementary Figure S15: Comparative $^1$H NMR spectra of seasonal variation.** Comparison of $^1$H NMR spectra of leaves samples of *A. scholaris* from each month of a year

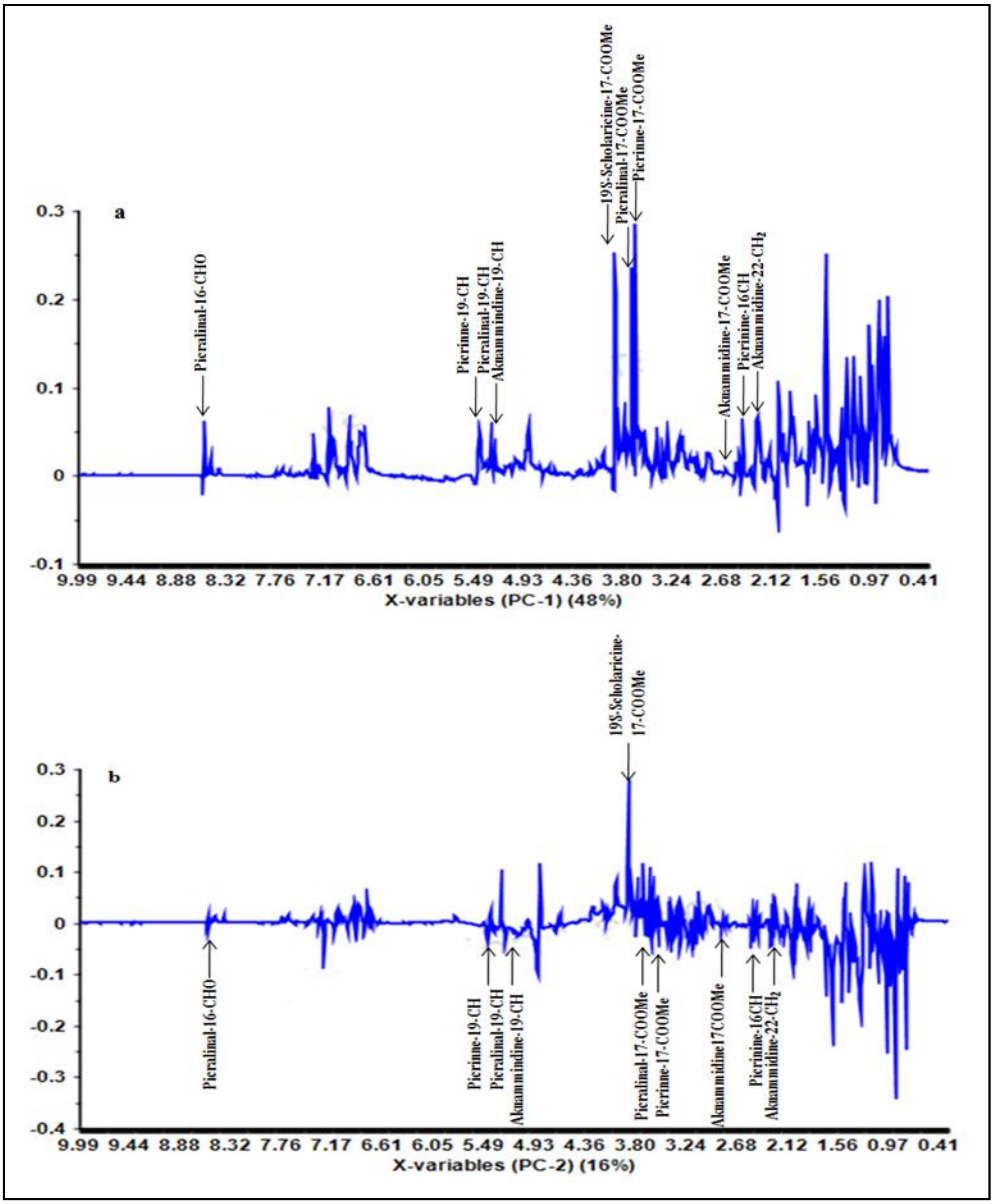


**Supplementary Figure S16: Loading derived from PCA of seasonal variation data.** Loading plots for (a) principal Component 1 (PC-1) and (b) principal component 2 (PC-2), of *A. scholaris* leaves sample of a whole year, showing variability of the alkaloids in PC analysis

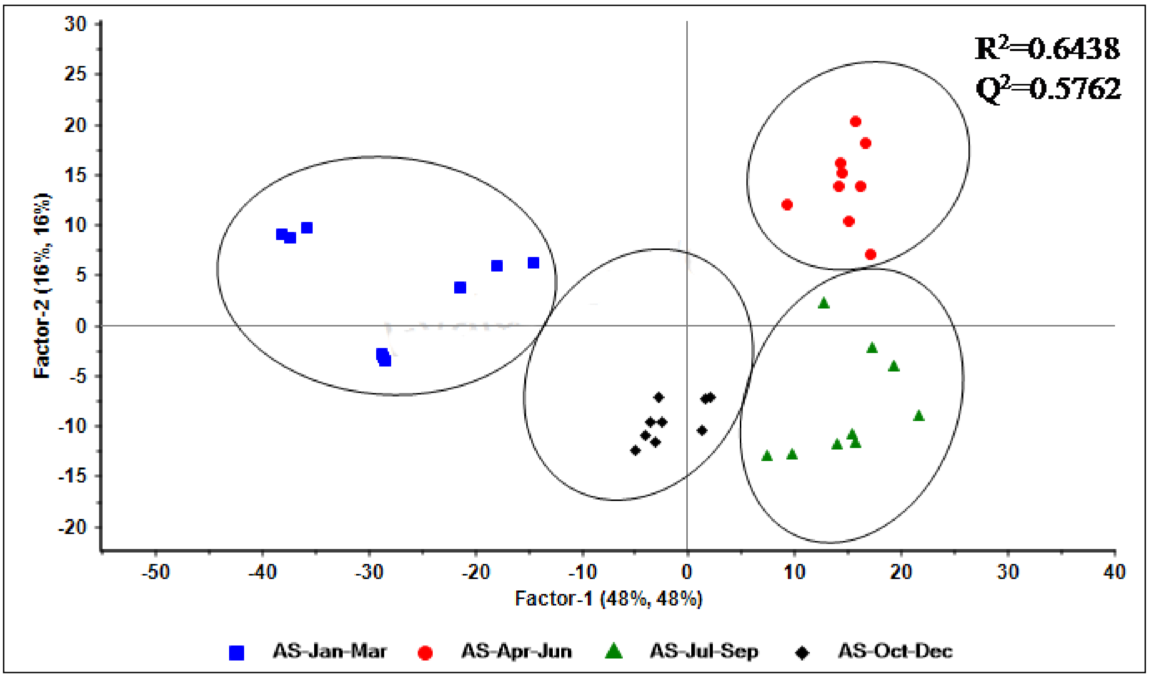


**Supplementary Figure S17: PLS DA of seasonal variation in group of 4 months.** PLS-DA 2D scatter scores plot of $^1$H NMR spectra of *A. scholaris*, showed four major group separation (a) AS-Jan-Mar, (b) AS-Apr-Jun, (c) Jul-Sep and (d) Oct-Dec along with $Q^2$ predictive ability and $R^2$ statistical validity

**Tables:**

**Supplementary Table S1: Concentration of alkaloids in plant parts.** Concentration of alkaloids expressed as mean±SEM of µg/g of all samples obtained from different parts of *A. scholaris*. Note: ND stands for not detected

| Alkaloids | Chemical Shift (ppm) | AS-FL | AS-FR | AS-L | AS-S | AS-TB |
|---|---|---|---|---|---|---|
| **Picrinine** | **3.65** | 67.56± 0.0823 | 73.41± 0.3292 | 54.92± 0.9538 | 4.462± 0.0475 | 16.30± 0.0453 |
| **Picralinal** | **8.56** | 28.75± 1.621 | 38.13± 0.1472 | 18.52± 0.2673 | 2.870± 0.0707 | ND |
| **Akuammidine** | **2.94** | 3.31± 0.2615 | 13.95± 0.0857 | 3.702± 0.0784 | 1.355± 0.055 | 0.2170± 0.0130 |
| **19 *S* Scholaricine** | **3.88** | 29.12± 0.4826 | 41.88± 0.4344 | 40.94± 0.5480 | 3.953± 0.1053 | 27.92± 0.1733 |

| | | | | | | |
|---|---|---|---|---|---|---|
| **19,20 *E* Vallesamine** | **3.75** | 29.01± 0.2083 | 35.99± 0.3608 | ND | 1.763± 0.0164 | 6.983± 0.0430 |
| **N$^b$-Demethylalsto-gustine *N*-Oxide** | **3.80** | ND | ND | ND | ND | 2.296± 0.1188 |
| **N$^b$-Demethyl-alstogustine** | **3.83** | ND | ND | ND | 1.897± 0.0191 | 20.16± 0.2164 |
| **Echitamine** | **3.73** | ND | ND | ND | ND | 14.21± 1.123 |

**Table S2: Significant difference between plant parts.** The statistical significance for alkaloids, determined by Student's t-test. NS, not significant; ND, not determined; A, AS-FL; B, AS-FR; C, AS-L; D, AS-S; E, AS-TB. The statistical significance is at *$p \leq 0.05$ and the most statistically significant at **$p \leq 0.01$

| Alkaloids | A vs B | A vs C | A vs D | A vs E | B vs C | B vs D | B vs E | C vs D | C vs E | D vs E |
|---|---|---|---|---|---|---|---|---|---|---|
| **Picrinine** | ** | ** | ** | ** | ** | ** | ** | ** | NS | ** |
| **Picralinal** | ** | ** | ** | ND | ** | ** | ND | ** | ND | ND |
| **Akuammidine** | ** | NS | ** | ** | ** | * | ** | ** | ** | ** |
| **19 *S* Scholaricine** | ** | ** | * | NS | NS | ** | ** | * | ** | ** |
| **19,20 *E* Vallesamine** | ** | ND | ND | ** | ND | ** | ** | ND | ND | ** |
| **N$^b$-demethyl alstogustine** | ND | ND | ND | ND | ND | ND | ND | ND | ND | ** |

**Table S3: Average concentration of alkaloids in group of 4 months.** Average concentration of alkaloids, expressed as mean values ±SE of µg/g of all samples from one year. Samples divided into four groups (a) AS (Jan-Mar), (b) AS (Apr-Jun), (c) AS (Jul-Sep) and (d) AS (Oct-Dec)

| Alkaloids | Chemical Shift (ppm) | AS (Jan-Mar) | AS (Apr-Jun) | AS (Jul-Sep) | AS (Oct-Dec) |
|---|---|---|---|---|---|
| **Picrinine** | **3.65** | 86.54± 8.15 | 125.44± 10.15 | 181.45± 0.9948 | 80.10± 5.577 |
| **Picralinal** | **8.56** | 32.31± 9.063 | 100.83± 1.408 | 127.40± 19.06 | 54.63± 5.490 |
| **Akuammidine** | **2.94** | 8.998± 9.036 | 23.64± 3.318 | 12.91± 0.9650 | 14.73± 0.2745 |
| **19 *S* Scholaricine** | **3.88** | 55.63± 13.16 | 178.08± 19.88 | 115.71± 8.254 | 28.89± 18.75 |

**Table S4: Significant difference in concentration between group of 4 months.** The statistical significance for alkaloids was determined by Student's t-test. NS for not significant, A, AS (Jan-Mar); B, AS (Apr-Jun); C, AS (Jul-Sep); D, AS (Oct-Dec). Numbers of subjects for per group were four. The statistical significance showed at * $p \leq 0.05$ and most significant at ** $p \leq 0.01$

| Alkaloids | A vs B | A vs C | A vs D | B vs C | B vs D | C vs D |
|---|---|---|---|---|---|---|

| | | | | | | |
|---|---|---|---|---|---|---|
| **Picrinine** | * | ** | NS | ** | ** | ** |
| **Picralinal** | ** | ** | NS | NS | ** | * |
| **Akuammidine** | NS | NS | NS | NS | ** | NS |
| **19 *S* Scholaricine** | ** | ** | NS | ** | ** | ** |
